# LPAR2 correlated with different prognosis and immune cell infiltration in head and neck squamous cell carcinoma and kidney renal clear cell carcinoma

**DOI:** 10.1186/s41065-022-00229-w

**Published:** 2022-03-04

**Authors:** Kai Sun, Ri-xin Chen, Jing-zhang Li, Zhan-xiong Luo

**Affiliations:** Department of Oncology, Liuzhou People’s Hospital, Guangxi Zhuang Autonomous Region, Liuzhou, 545001 China

**Keywords:** Head and neck squamous cell carcinoma, Kidney renal clear cell carcinoma, Prognosis, LPAR2, Tumor immune infiltration

## Abstract

**Background:**

Lysophosphatidic acid (LPA) and its receptors play a key role in regulating cancer progression. Upregulation of LPA receptor 2 (*LPAR2*) plays a role in carcinogenesis; however, the exact role of *LPAR2* in tumors remains elusive. This study aims to explore the correlation between LPAR2 expression with tumor prognosis and immune infiltration in pan-cancers.

**Materials and methods:**

The expression of *LPAR2* in pan-cancers was analyzed using the Online Cancer Microarray Database (Oncomine), Tumor Immune Estimation Resource (TIMER), and UALCAN databases. The effects of *LPAR2* on the clinical prognosis in pan-cancer were examined using the Kaplan–Meier plotter (KM plotter) as well as Gene Expression Profiling Interactive Analysis (GEPIA), UALCAN, and Human Protein Atlas (HPA) databases. Moreover, the R software program was applied for validation of expression and prognostic value of *LPAR2* in tumor patients in the Cancer Genome Atlas (TCGA) dataset and the Gene Expression Omnibus (GEO) database. The relationship between the expression level of *LPAR2* and the clinical and molecular criteria of head and neck squamous cell carcinoma (HNSC) and kidney renal clear cell carcinoma (KIRC) was analyzed using UALCAN, whereas the relationship between LPAR2 expression and prognosis in patients with HNSC and KIRC with different clinical characteristics was examined using the KM plotter. Furthermore, the correlation between *LPAR2* expression and tumor immune infiltration was examined using TIMER. The correlation between LPAR2 expression and gene markers of tumor immune infiltrates was analyzed using TIMER and GEPIA. In addition, the cBioPortal for Cancer Genomics was used to calculate the mutations, methylations, and altered neighbor genes of *LPAR2*.

**Results:**

The expression of *LPAR2* was significantly correlated with the outcome of multiple types of cancer, especially HNSC and KIRC. Furthermore, high expression of *LPAR2* was significantly associated with various immune markers in the immune cell subsets of HNSC and KIRC.

**Conclusions:**

High expression of *LPAR2* plays significantly different prognostic roles in HNSC and KIRC possibly owing to its association with different immune markers. *LPAR2* is correlated with tumor immune cell infiltration and is a valuable prognostic biomarker for HNSC and KIRC. However, further experiments are required to validate these findings.

**Supplementary Information:**

The online version contains supplementary material available at 10.1186/s41065-022-00229-w.

## Introduction

Lysophosphatidic acid (LPA, 1-acyl-2-hemolytic-sn-glycerin-3-phosphate) is a bioactive glycerophosphatidic acid, which is a naturally occurring lysophospholipid and is abundantly found in the human body [1, 2]. Lipopolysaccharides, lysophosphatidylethanolamine, and lysophosphatidylcholine are hydrolyzed by autotaxin to produce LPA in plasma, serum, and adipocytes [3]. LPA serves as a growth factor by activating distinct high-affinity G protein-coupled receptors (GPCRs), which promote the growth, differentiation, migration, division, and survival of various cell types [4, 5]. LPA has several GPCRs known as LPA receptors (LPARs) [6]. According to their homology, LPARs can be divided into six types, namely, *LPAR1, LPAR2, LPAR3, LPAR4, LPAR5,* and *LPAR6*, which can be grouped into two subfamilies, namely, endothelial differentiation gene (EDG) family (*LPAR1–3*) and purinergic receptor family (*LPAR4–6*) [7]. LPARs contain seven transmembrane domains, three intracellular loops, and three extracellular loops [8]. The LPAR signaling pathway produces different results in different environments and cell types, and at least two Gα subunits are involved (Gαq/11, Gα12/13, Gαi/o, and GαS) that activate different downstream pathways [9, 10]. Several signaling pathways, such as RhoA, phospholipase C, PI3K/PAK1/ERK, Ras–Raf–MEK–ERK, and Rac pathways, are activated by Gαq/11, Gα12/13, Gαi/o, and GαS [9, 11]. Owing to the presence of similar G protein types, the six LPARs perform similar biological functions [12]. Multiple studies have revealed the key roles of LPA and LPARs in various cancer tissues, such as in breast, lung, liver, pancreatic, ovarian, and thyroid cancers and neuroblastoma [13, 14].

Although many studies have described the expression and function of *LPAR1* and *LPAR3* in several tumors, studies on *LPAR2* are limited [15]. Several studies have reported that *LPAR2* is aberrantly expressed in several tumors, including breast, colorectal, kidney, and pancreatic cancers [16–19], and promotes robust activation of RhoA to mediate cell migration [20]. A recent study demonstrated that *LPAR2* regulated cell–cell adhesion of neural crest cells by internalizing N-cadherin downstream of *LPAR2 *[21]. In addition, a study reported that *LPAR2* is significantly associated with LPA-induced expression of interleukin (IL)-6 and IL-8, which promoted breast cancer progression [22, 23]. However, the mechanism of action of *LPAR2* in tumors appears diverse and remains unclear [24].

**Fig. 1:**
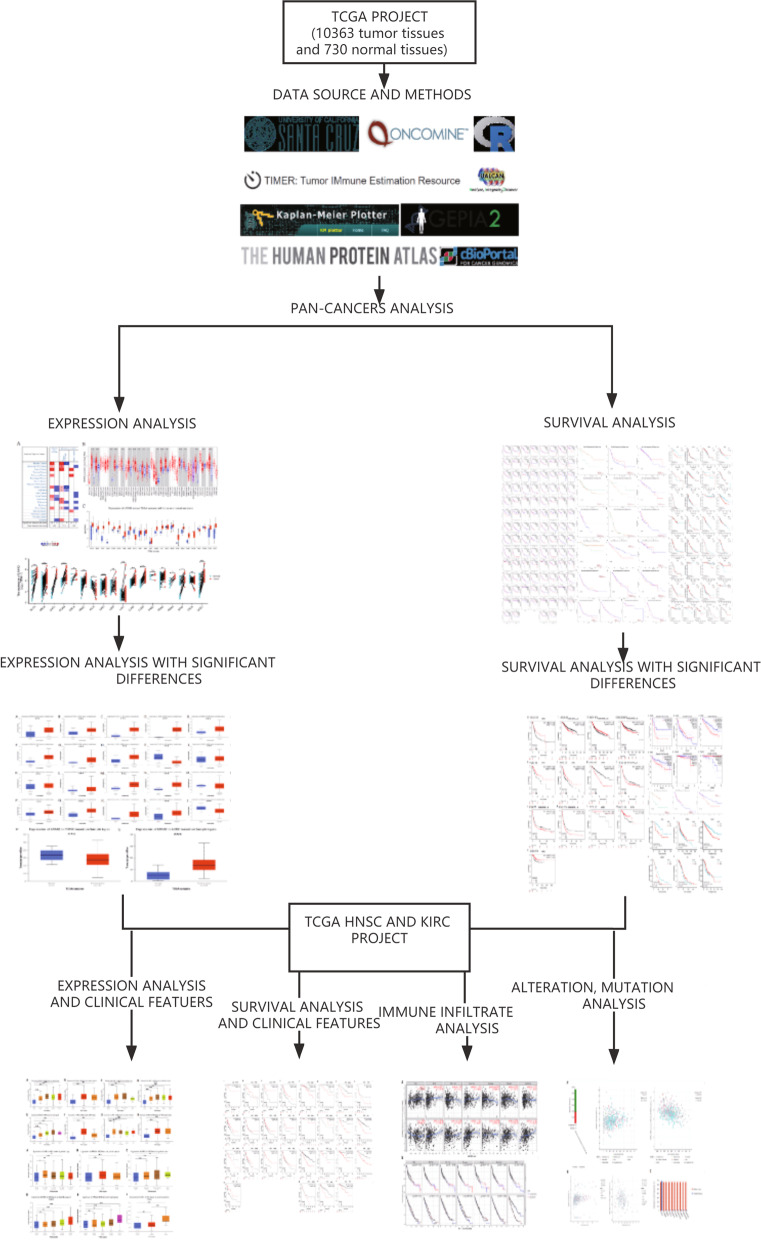
Analysis workflow of this research

In this study, we systematically investigated the expression of *LPAR2* and its relationship with pan-cancer prognosis using the Oncomine, TIMER, UALCAN, GEPIA, KM plotter and HPA databases, as well as expression and survival analysis of *LPAR2* in the TCGA and GEO data was validated by R software. Furthermore, we examined the relationship between *LPAR2* expression and the clinical and molecular criteria of HNSC and KIRC using UALCAN. Subsequently, we investigated the relationship between *LPAR2* expression and the prognosis of patients with HNSC and KIRC with different clinical characteristics using the KM plotter. In addition, we analyzed the correlation between *LPAR2* and tumor-infiltrating immune cells in the microenvironment of pan-cancer using TIMER and GEPIA. Lastly, we used the cBioPortal for Cancer Genomics online tool to analyze the alterations, mutations, methylations, and pathways of *LPAR2*. Therefore, in this study, we demonstrated a potential mechanism of action of *LPAR2*, examined the prognostic role of *LPAR2* in HNSC and KIRC, and identified *LPAR2* as a key factor in regulating the immune microenvironment of HNSC and KIRC. The overall design and workflow of this study is presented in Fig. 1.

## Results

### Assessment of *LPAR2* expression in different cancers and normal tissues

**Fig. 2:**
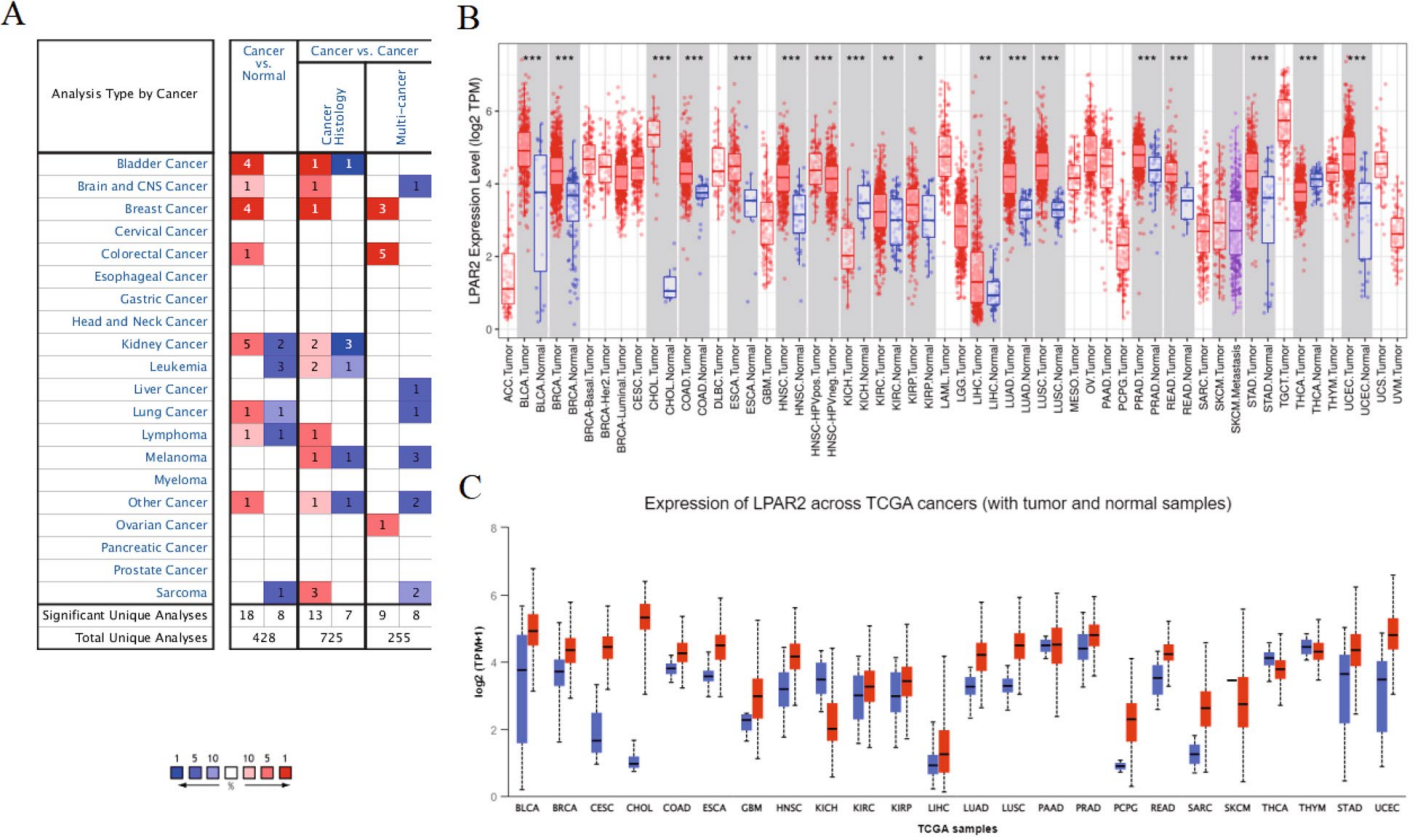
**A** The transcription levels of LPAR2 in different cancers (ONCOMINE). **B** LPAR2 expression levels in different tumor types from TCGA database were determined by TIMER. C LPAR2 expression levels in different tumor types from TCGA database were determined by UACLAN. *P < 0.05, **P < 0.01, ***P < 0.001

**Fig. 3:**
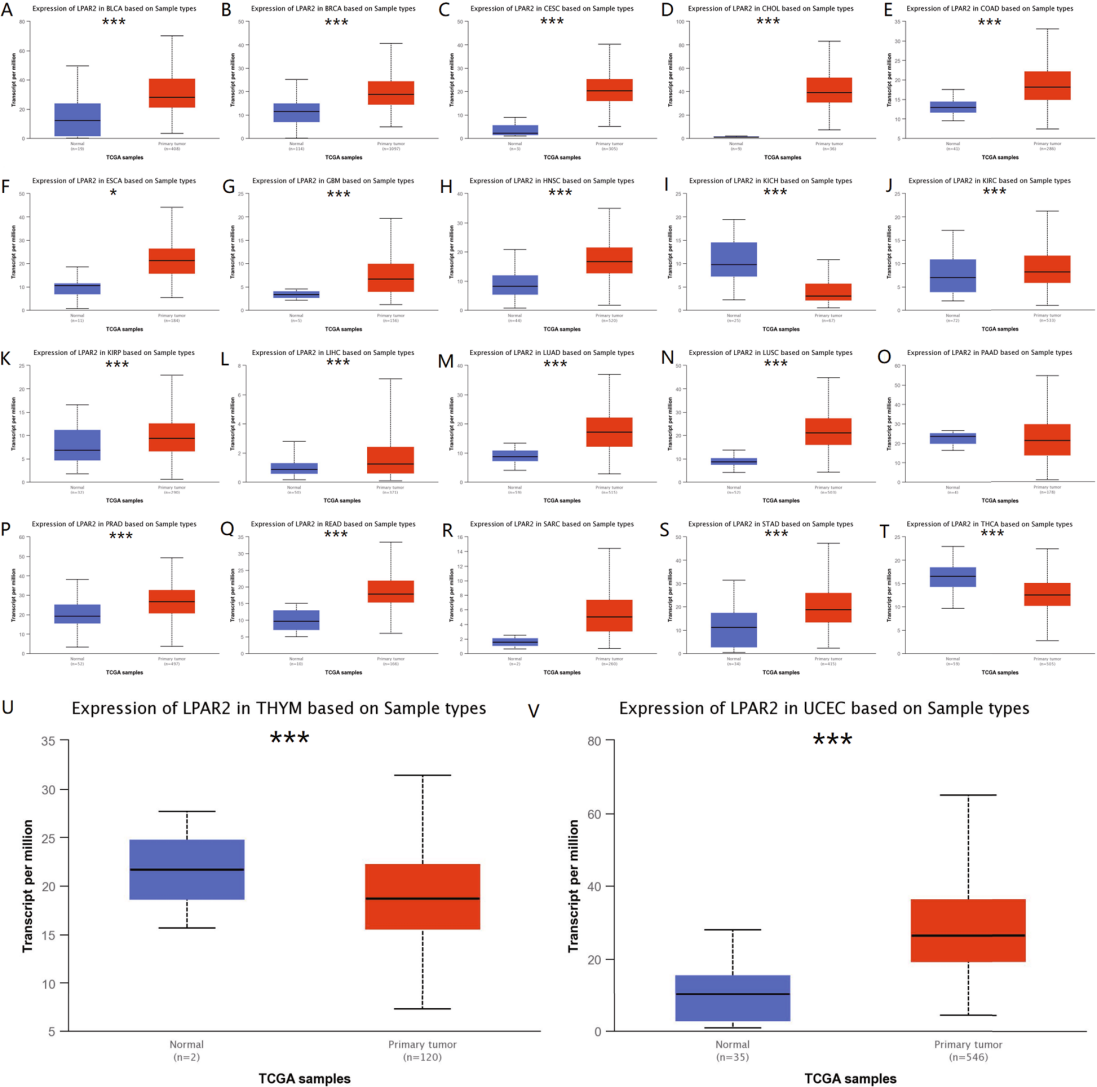
LPAR2 mRNA expression levels in different tumor types from TCGA database were determined by UCLAN. *P < 0.05, **P < 0.01, ***P < 0.001

**Fig. 4:**
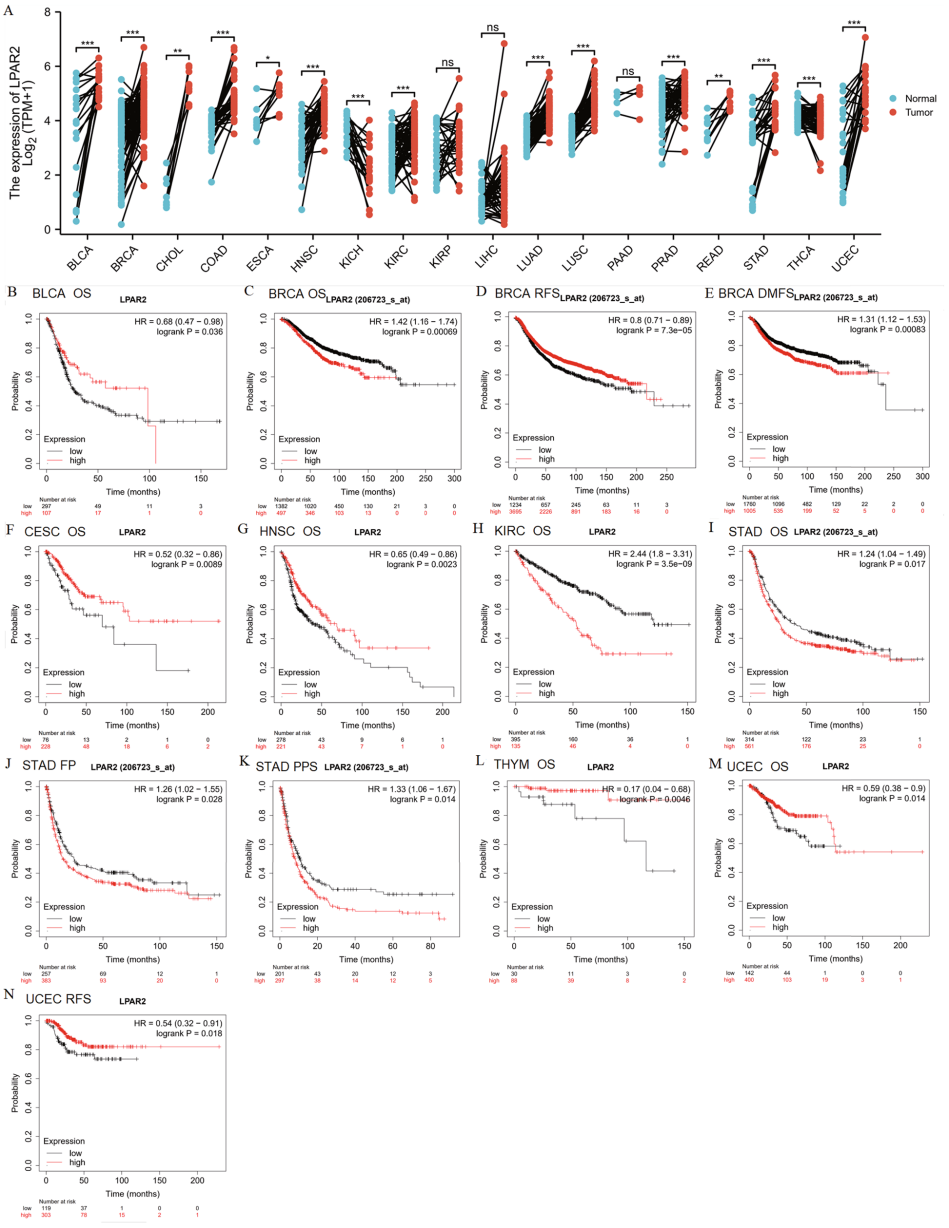
A LPAR2 mRNA expression levels in different tumor types from TCGA database. B-N Kaplan–Meier survival curves comparing the high and low expression of LPAR2 in different types of cancers in the KM plotter databases. *P < 0.05, **P < 0.01, ***P < 0.001. Abbreviations: OS, overall survival; DFS, disease-free survival; RFS, relapse-free survival; DSS, disease-specific survival; DMFS, distant metastasis-free survival; FP, first progression; HR: hazard ratio

On analyzing the mRNA expression levels of *LPAR2* in pan-cancer and normal tissues using Oncomine, we found that *LPAR2* expression was higher in bladder, brain and central nervous system (CNS), breast, colorectal, kidney, and lung cancers and lymphoma than in normal control tissues (Fig. 2A). However, *LPAR2* expression was lower in kidney cancer, leukemia, lung cancer, lymphoma and sarcoma tissues than in normal control tissues (Fig. 2A). Table 1 summarizes the detailed findings of specific tumor types. Furthermore, we assessed differences in *LPAR2* expression in pan-cancer using the TIMER databases and found that *LPAR2* expression was significantly higher in bladder urothelial carcinoma (BLCA), breast invasive carcinoma (BRCA), cholangiocarcinoma (CHOL), colon adenocarcinoma (COAD), esophageal carcinoma (ESCA), HNSC, KIRC, liver hepatocellular carcinoma (LIHC), lung adenocarcinoma (LUAD), lung squamous cell carcinoma (LUSC), prostate adenocarcinoma (PRAD), rectum adenocarcinoma (READ), stomach adenocarcinoma (STAD), and uterine corpus endometrial carcinoma (UCEC) than in the adjacent normal tissues (Fig. 2B). However, *LPAR2* expression was significantly lower in kidney chromophobe (KICH) and thyroid carcinoma (THCA) than in the adjacent normal tissues (Fig. 2B). Subsequently, we examined *LPAR2* expression using UALCAN and found that the mRNA expression levels of *LPAR2* were significantly higher in BLCA, BRCA, cervical squamous cell carcinoma and endocervical adenocarcinoma (CECS), glioblastoma multiforme (GBM), HNSC, KIRC, kidney renal papillary cell carcinoma (KIRP), LIHC, LUAD, LUSC, PRAD, READ, STAD, and UCEC than in normal control tissues (Figs. 2C, 3). However, *LPAR2* expression was significantly lower in KICH and THCA than in normal control tissues (Fig. 3). Differences in *LPAR2* expression between tumors and normal adjacent tissue samples are demonstrated in Fig. 1C. To validate these results, we used R software to analyze expression of LPAR2 in pan-cancers via the TCGA databases (Fig. 4A). As a result, we observed the same trend in the expression of LPAR2 in pan-cancers (Fig. 4A).

**Table 1:** The significant changes of LPAR2 expression in cancers vs normal tissue in oncomine database

Cancer	Cancer type	*P*-value	Fold change	Rank (%)	Sample	Reference
Bladder Cancer	Superficial Bladder Cancer vs. Normal	4.02E-23	5.967	1%	41	Sanchez-Carbayo Bladder 2
	Infiltrating Bladder Urothelial Carcinoma vs. Normal	5.43E-11	2.342	3%	367	Sanchez-Carbayo Bladder 2
	Superficial Bladder Cancer vs. Normal	1.35E-7	1.582	3%	375	Dyrskjot Bladder 3
	Superficial Bladder Cancer vs. Normal	8.31E-6	1.504	4%	652	Lee Bladder
Brain and CNS Cancer	Anaplastic Astrocytoma vs. Normal	4.10E-5	2.255	8%	1521	Sun Brain
Breast cancer	Mixed Lobular and Ductal Breast Carcinoma vs. Normal	3.13E-9	1.889	1%	50	TCGA Breast
	Invasive Lobular Breast Carcinoma vs. Normal	1.24E-9	1.791	8%	1551	TCGA Breast
	Invasive Breast Carcinoma vs. Normal	1.07E-11	1.756	10%	1942	TCGA Breast
	Medullary Breast Carcinoma vs. Normal	1.26E-7	1.619	10%	1742	Curtis Breast
Colorectal Cancer	Rectal Adenoma vs. Normal	1.09E-6	2.735	3%	549	Sabates-Bellver Colon
Kidney Cancer	Papillary Renal Cell Carcinoma vs. Normal	2.40E-13	1.532	2%	220	Jones Renal
	Chromophobe Renal Cell Carcinoma vs. Normal	4.03E-6	1.673	6%	663	Jones Renal
	Renal Oncocytoma vs. Normal	5.97E-9	1.969	6%	650	Jones Renal
	Clear Cell Renal Cell Carcinoma vs. Normal	1.78E-10	1.688	7%	801	Jones Renal
	Renal Pelvis Urothelial Carcinoma vs. Normal	5.02E-6	1.870	8%	933	Jones Renal
	Clear Cell Renal Cell Carcinoma vs. Normal	4.38E-19	-2.100	4%	437	Jones Renal
	Papillary Renal Cell Carcinoma vs. Normal	9.72E-13	-1.613	4%	480	Jones Renal
Leukemia	T-Cell Acute Lymphoblastic Leukemia vs. Normal	3.28E-9	-8.139	2%	110	Andersson Leukemia
	Acute Myeloid Leukemia vs. Normal	1.17E-9	-6.503	2%	181	Andersson Leukemia
	B-Cell Acute Lymphoblastic Leukemia vs. Normal	8.15E-8	-9.264	9%	859	Andersson Leukemia
Lung caner	Lung Adenocarcinoma vs. Normal	2.50E-14	1.623	4%	612	Selamat Lung
	Small Cell Lung Carcinoma vs. Normal	8.31E-5	-4.067	9%	741	Bhattacharjee Lung
Lymphoma	Diffuse Large B-Cell Lymphoma vs. Normal	2.31E-5	1.551	6%	1085	Brune Lymphoma
	Unspecified Peripheral T-Cell Lymphoma vs. Normal	6.08E-12	-1.797	2%	314	Piccaluga Lymphoma
Other cancer	Testicular Seminoma vs. Normal	7.73E-8	1.859	3%	284	Sperger Others
Sarcoma	Gastrointestinal Stromal Tumor vs. Normal	3.90E-10	-4.256	2%	269	Cho Gastric

### Relationship between *LPAR2* expression and prognosis in patients with cancer

**Fig. 5:**
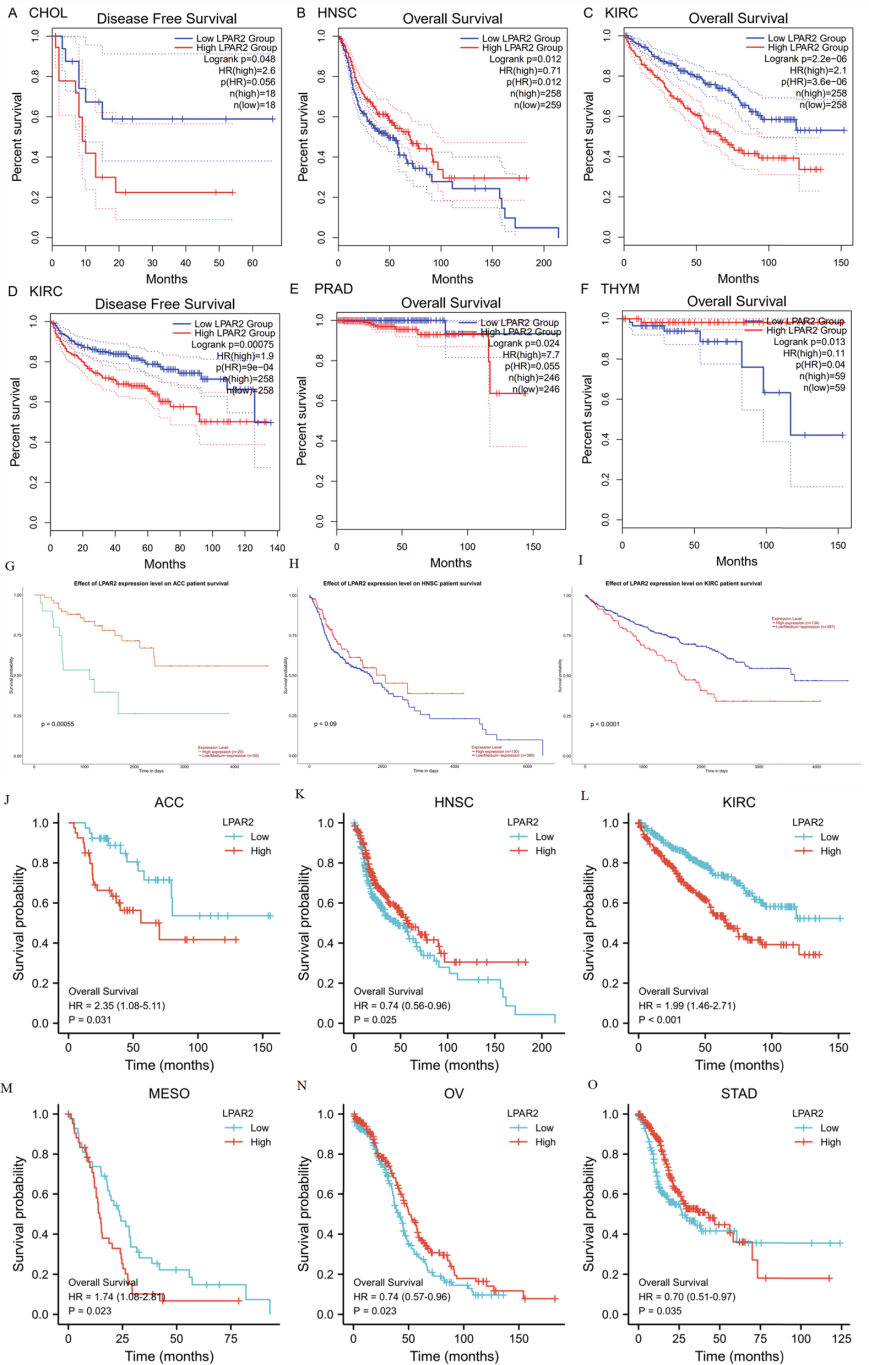
**A**-**F** Prognostic analysis of LPAR2 mRNA expression levels in different tumor types in GEPIA databases. G-H Correlation between LPAR2 gene expression and survival prognosis of cancers in UALCAN databases. **J**-**O** Correlation between LPAR2 gene expression and OS of cancers in TCGA. Abbreviations: OS, overall survival; DFS, disease-free survival; RFS, relapse-free survival; DSS, disease-specific survival. DMFS, distant metastasis-free survival

We used KM plotter to determine the correlation between *LPAR2* expression and the survival of patients with pan-cancer and those with normal tissues (Figure S1). A significant correlation was observed between *LPAR2* expression and prognosis in BLCA, BRCA, CESC, HNSC, KIRC, STAD, THYM, and UCEC (Fig. 4B-N). In addition, we found that high *LPAR2* expression was significantly associated with a worse prognosis in patients with BRCA (overall survival [OS], HR = 1.42 [1.16 − 1.74], *P* = 0.00069; distant metastasis-free survival [DMFS], HR = 1.31 [1.12 − 1.53], *P* = 0.00083), STAD (OS, HR = 1.24 [1.04 − 1.49], *P* = 0.017; first progression [FP], HR = 1.26 [1.02–1.55], *P* = 0.028; and post-progression survival [PPS], HR = 1.33 [1.06 − 1.67], *P* = 0.014) and KIRC (OS, HR = 2.44 [1.8 − 3.31], *P* = 3.5e − 09) (Fig. 4C, E, H–K). On the contrary, high *LPAR2* expression was associated with improved prognosis in patients with BLCA (OS, HR = 0.68 [0.47 − 0.98], *P* = 0.036), CESC (OS, HR = 0.52 [0.32 − 0.86], *P* = 0.0089), HNSC (OS, HR = 0.65 [0.49 − 0.86], *P* = 0.0023), TYHM (OS, HR = 0.17 [0.04 − 0.68], *P* = 0.0046), UCEC (OS, HR = 0.59 [0.38 − 0.9], *P* = 0.014, RFS, HR = 0.54 [0.32–0.91], *P* = 0.018) and BRCA (RFS, HR = 0.8 [0.71–0.89], *P* = 7.3e − 05) (Fig. 4B, F, G, L, M, N). However, no significant correlation was observed between the mRNA expression levels of *LPAR2* and the prognosis of other cancers (Figure S1). Furthermore, we assessed the relationship between *LPAR2* expression and the prognosis of multiple cancers using GEPIA (Figure S2) and found that high mRNA expression of *LPAR2* was associated with a worse prognosis in patients with KIRC (OS, HR = 2.1, *P* = 3.6e − 06; disease-free survival [DFS], HR = 1.9, *P* = 9e − 04), PRAD (OS, HR = 7.7, *P* = 0.024), and CHOL (DFS, HR = 2.6, *P* = 0.048) (Fig. 5A, C–E). Furthermore, high mRNA expression of *LPAR2* was correlated with better OS in patients with HNSC (HR = 0.71, *P* = 0.012) and THYM (HR = 0.11, *P* = 0.013) (Fig. 5B, F). However, it was not significantly correlated with OS and DFS in patients with BRCA (OS, HR = 0.85, *P* = 0.49; DFS, HR = 0.74, *P* = 0.29) and other tumors (Figure S2). In UALCAN databases, we found that higher expression of LPAR2 was associated with improved prognosis in patients with ACC (*P* = 0.00055), as well as with worse prognosis with KIRC (*P* < 0.0001) (Fig. 5G, I). Upregulation of LPAR2 might be correlated with good prognosis in HNSC patients, but this correlation was not statistically significant (Fig. 5H). Nevertheless, in UACLAN, no significant correlation was observed between LPAR2 expression and the prognosis of other cancers (Figure S3).

Likewise, to validate these results, survival analysis of LPAR2 in pan-cancers of the TCGA databases was performed using the survival package via R software (Figure S4). Our results indicated that high expression level of *LPAR2* was significantly associated with a worse OS in patients with ACC (HR = 2.35[1.08 − 5.11], *P* = 0.031), KIRC (HR = 1.99 [1.46 − 2.71], *P* < 0.001) and MESO (OS, HR = 1.74 [1.08 − 2.81], *P* = 0.023) (Fig. 5 J, L, M). On the other hand, high LPAR2 expression was associated with improved prognosis in patients with HNSC (HR = 0.74[0.56 − 0.96], *P* = 0.025), OV (HR = 0.74 [0.57 − 0.96], *P* = 0.023) and STAD (OS, HR = 0.70 [0.51 − 0.97], *P* = 0.035) (Fig. 5 K, N, O).

**Fig. 6:**
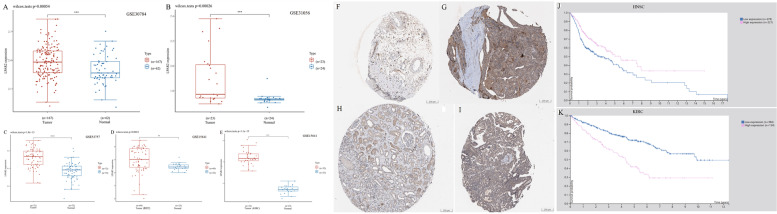
**A**-**E** Relative mRNA expression of LPAR2 in HNSC and KIRC and paired normal tissues from GEO database. (**A**: in GSE30784; **B**: in GSE31065; **C**: in GSE53757; **D**, **E**: in GSE15641.) **F**-**I** Representative immunohistochemistry images of different LPAR2 in HNSC and KIRC tissues and corresponding normal tissues from the human protein atlas database (HPA). (**F**: Oral normal tissue; **G**: Head-Neck Squamous cell carcinoma tissue; **H**: Kidney normal tissue; **I**: Kidney renal clear cell carcinoma tissue.) **J**-**K** Correlation between LPAR2 gene expression and survival prognosis of in HNSC and KIRC from HPA. (**J**: OS OF HNSC; **K**: OS OF KIRC.). *P < 0.05, **P < 0.01, ***P < 0.001

Taken together, the combination of OS, RFS, DFS and DMFS, and concern of bias, our findings illustrated the expression levels and prognostic value of *LPAR2* in several types of cancers, especially HNSC and KIRC, suggesting that high *LPAR2* expression plays significantly different prognostic roles in HNSC and KIRC. Thus, we performed *LPAR2* expression analyses and survival analyses in HNSC and KIRC using GEO databases in the end. Results of differential expression analysis showed that LPAR2 expression was significantly higher in HNSC and KIRC than in normal control tissues in GSE30784, GSE31056, GSE53757 and GSE15641(*P* < 0.01) (Fig. 6A-E). However, survival analysis of GSE686, GSE65858, GSE167573 and GSE22541 showed that no further significant correlations were found between LPAR2 expression and the prognosis of HNSC and KIRC (*P* > 0.05) (Figure S5A-D). These inconsistencies might be due to limited sample sizes of HNSC and KIRC in GEO databases and differences in data collection methods as well as biases in methods of adjustment. Therefore, much further experimental validation is needed to investigate the link between the expression of *LPAR2* and prognosis in cancer patients with HNSC and KIRC as well as other kinds of cancers.

### Relationship between protein expression of *LPAR2* and prognosis in patients with HNSC and KIRC

After analyzing the mRNA expression of *LPAR2* and its relationship with the prognosis of patients with HNSC and KIRC, we investigated the protein expression of *LPAR2* and its correlation with the prognosis of patients with HNSC and KIRC using the HPA database. As demonstrated in Fig. 6 F-I, the protein expression of *LPAR2* was moderate in HNSC and KIRC tissues and low in the corresponding normal tissues. Relevant clinical data was shown in Table S1. Furthermore, according to the data obtained from the HPA, the relationship between the protein expression of *LPAR2* and prognosis was similar to that between the mRNA expression of *LPAR2* and prognosis. Moreover, high protein expression of *LPAR2* was associated with worse OS in patients with KIRC (*P* = 3.5e-9) but with improved OS in patients with HNSC (*P* = 0.0023) (Fig. 6 J–K). The related clinical data were exhibited in Table S2 and Table S3.

### Relationship between mRNA expression of *LPAR2* and clinical characteristics of patients with HNSC and KIRC

**Fig. 7:**
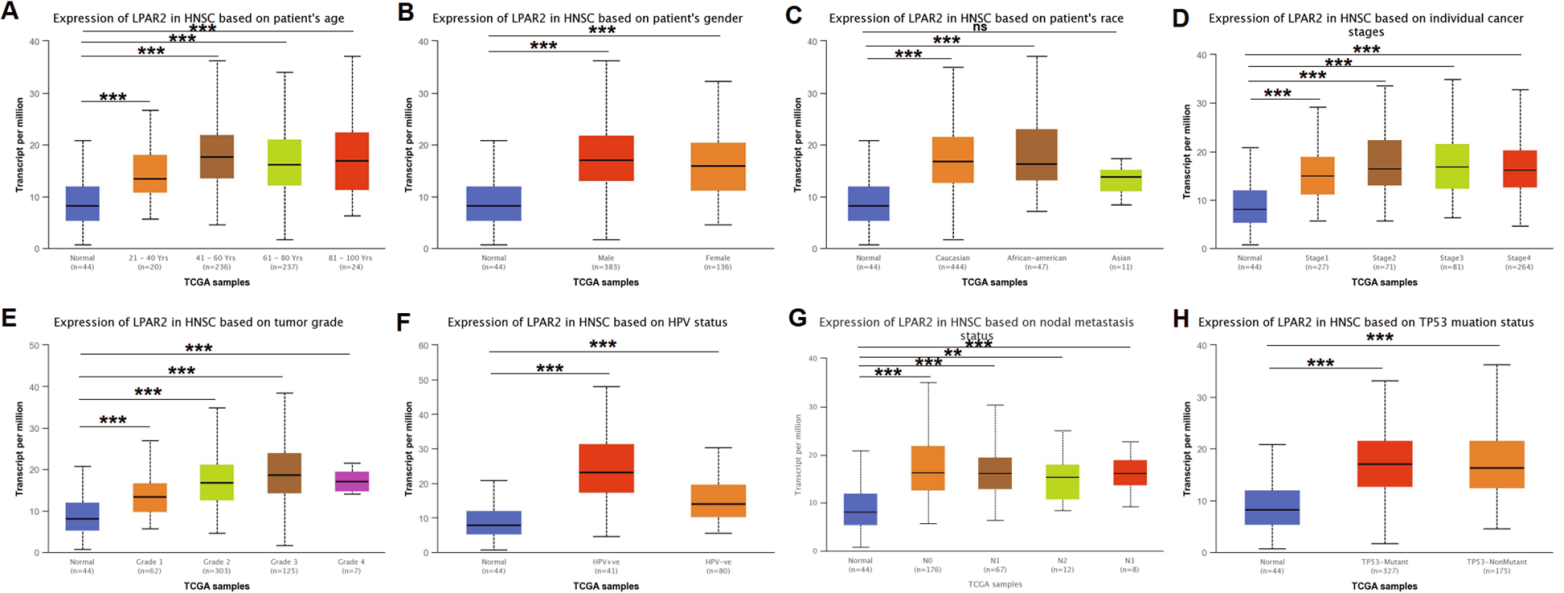
the relationship between the LPAR2 mRNA expression and clinical characteristics of HNSC patients from TCGA database in UCLAN(**A**-**H**). *P < 0.05, **P < 0.01, ***P < 0.001

Given that *LPAR2* expression plays significantly different prognostic roles in HNSC and KIRC, we used UALCAN and TCGA to examine the relationship between *LPAR2* expression and the clinical characteristics of patients with HNSC and KIRC. For the criterion of tumor stage, we found that *LPAR2* expression was significantly higher in patients with stage 1–4 HNSC than in patients in the control group (*P* < 0.001) (Fig. 7D). For the criterion of race, the mRNA expression of *LPAR2* was higher in the Caucasian and African–American patients with HNSC than in patients in the control group (*P* < 0.001); however, there was no significant difference in *LPAR2* expression between the Asian patients with HNSC and those in the control group (*P* > 0.05) (Fig. 7C). In addition, *LPAR2* expression was upregulated in both men and women with HNSC (*P* < 0.001) (Fig. 7B) in the age groups of 21–40 years (*P* < 0.001), 41–60 years (*P* < 0.001), 61–80 years, and 81–100 years (*P* < 0.001) (Fig. 7A). These findings suggested that the mRNA expression of *LPAR2* was significantly higher in patients with HNSC than in patients in the control group (*P* < 0.01 and *P* < 0.001, respectively), irrespective of tumor grade, HPV expression status, nodal metastasis status, and mutation status (Fig. 7E, F, G, H.).

**Fig. 8:**
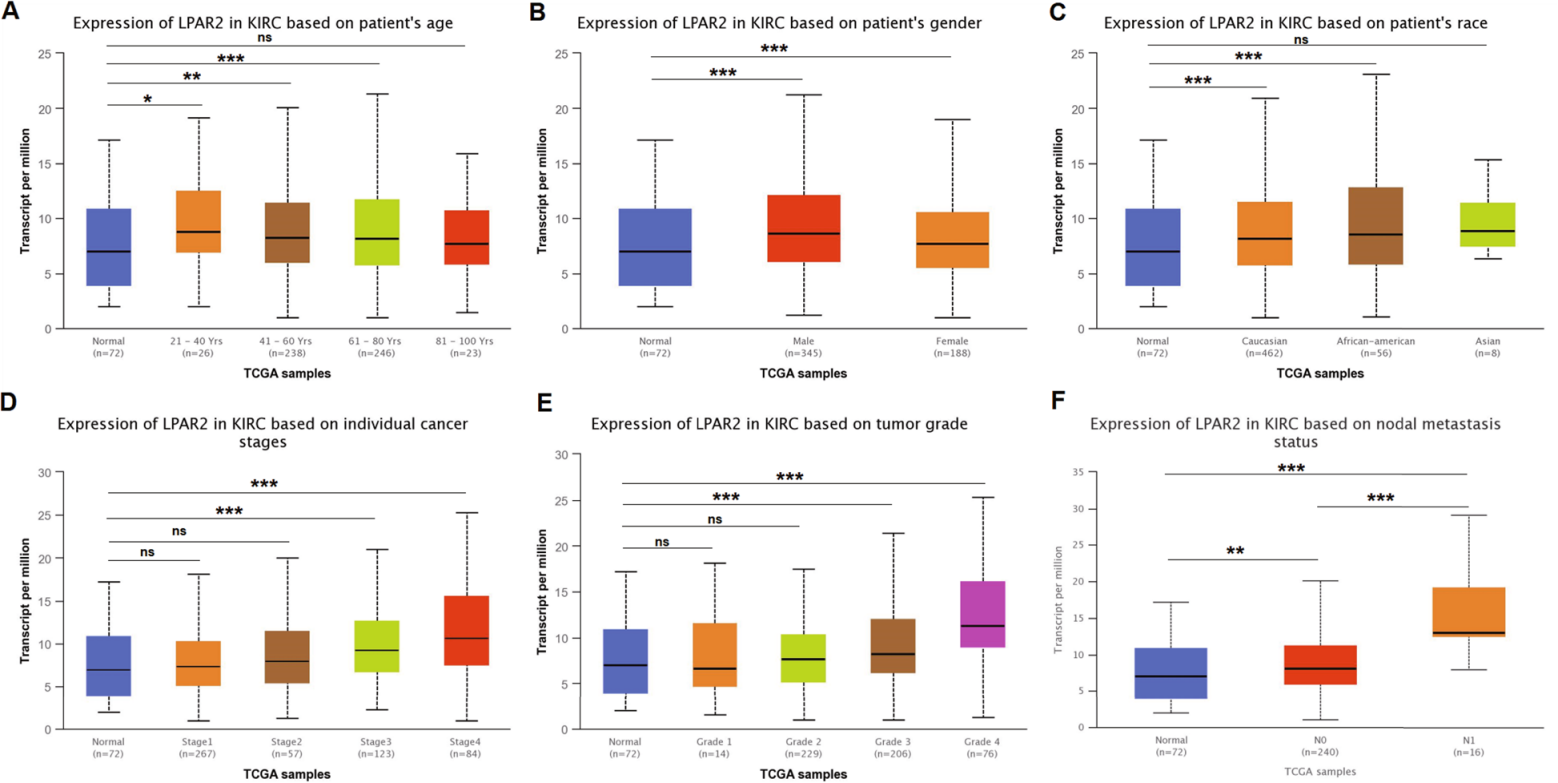
the relationship between the LPAR2 mRNA expression and clinical characteristics of KIRC patients from TCGA database in UCLAN. *P < 0.05, **P < 0.01, ***P < 0.001

In patients with KIRC, *LPAR2* expression was upregulated in patients with tumor stages 3 and 4 (*P* < 0.001) (Fig. 8D). However, there was no significant difference in LPAR2 expression between patients with tumor stages 1–2 and those in the control group (*P* > 0.05) (Fig. 8D). Similar to HNSC, *LPAR2* expression was significantly higher in the Caucasian and African–American patients with KIRC than in patients in the control group (*P* < 0.001); whereas there was no significant difference in *LPAR2* expression between the Asian patients with KIRC and those in the control group (*P* > 0.05) (Fig. 8C). In addition, *LPAR2* expression was upregulated in both men and women with KIRC (*P* < 0.001) (Fig. 8B). Meanwhile, we found that *LPAR2* expression was upregulated in patients with KIRC in the age groups of 21–40 years (*P* < 0.05), 41–60 years (*P* < 0.01), and 61–80 years (*P* < 0.001) but not in the age group of 81–100 years (*P* > 0.05) (Fig. 8A). Our findings also suggested that the mRNA expression of *LPAR2* was higher in patients with grade 3–4 KIRC than in patients in the control group (*P* < 0.001); nonetheless there was no significant difference between the mRNA expression of *LPAR2* in patients with grade 1–2 KIRC and those in the control group (*P* > 0.05) (Fig. 8E). Furthermore, the mRNA expression of *LPAR2* was higher in patients with node-positive KIRC than in patients with node-negative KIRC; however, it was higher in both node-positive and node-negative patients than in patients in the control group (*P* < 0.01 and *P* < 0.001, respectively) (Fig. 8F). These findings suggested that *LPAR2* expression was associated with tumor stage, tumor grade, and lymph node metastasis in patients with KIRC, and with race in patients with HNSC and KIRC.

### Relationship between mRNA expression of *LPAR2* and prognosis in patients with HNSC and KIRC with different clinical characteristics

**Fig. 9:**
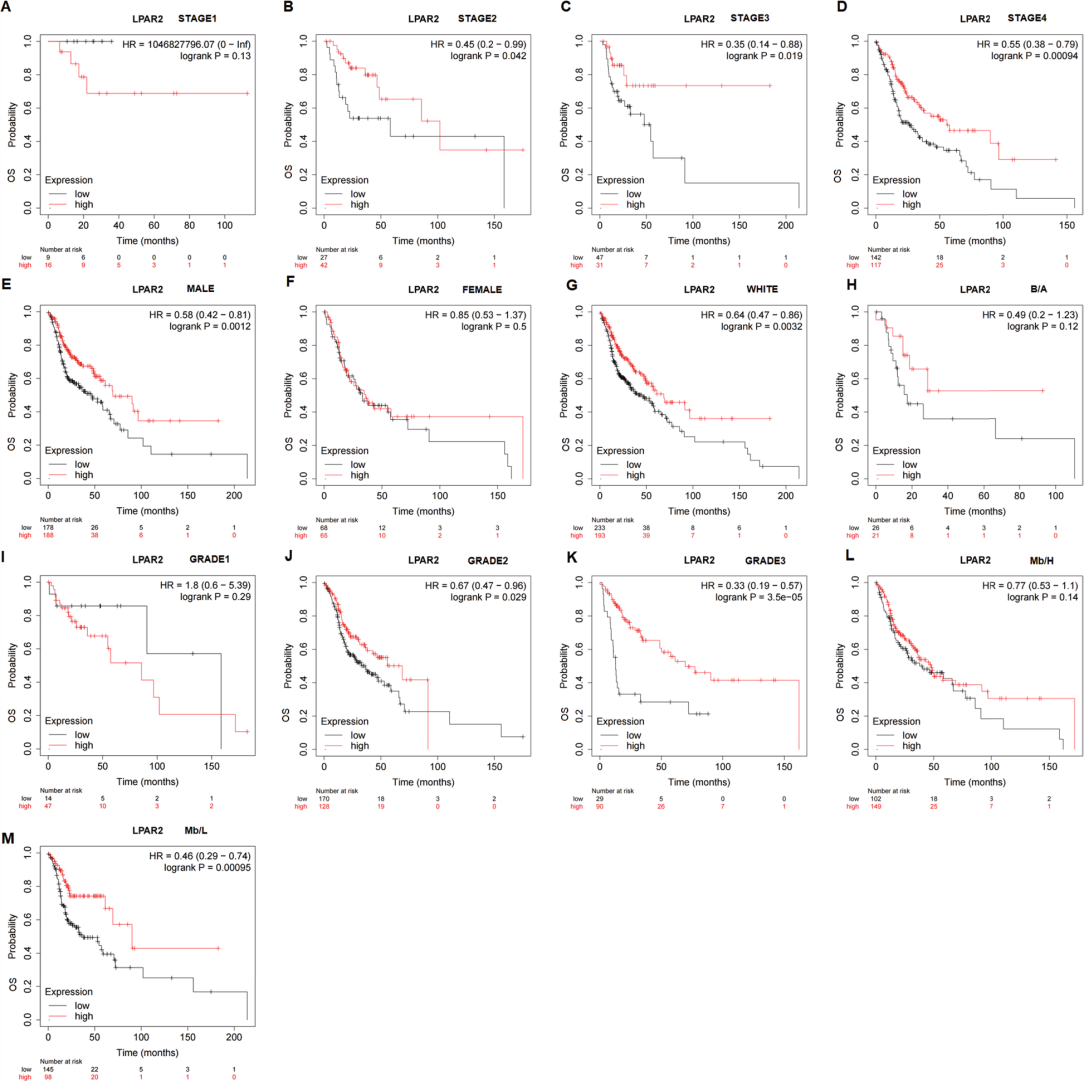
the relationship between the LPAR2 mRNA expression and prognosis in HNSC patients with different clinical characteristics in Kaplan–Meier plotter databases(A-M). Abbreviations: OS, overall survival; DFS, disease-free survival; RFS, relapse-free survival; DSS, disease-specific survival. DMFS, distant metastasis-free survival. Mb:H, Mutation burden high; Mb:L, Mutation burden low

For a better understanding of the mechanisms of *LPAR2* expression in HNSC and KIRC, we assessed the relationship between mRNA expression of *LPAR2* and prognosis in patients with HNSC and KIRC with different clinical characteristics in KM plotter. Higher mRNA expression of *LPAR2* was associated with better OS in HNSC tumor stage 2–4 (stage 2, HR = 0.45 [0.2–0.99], *P* = 0.042; stage 3, HR = 0.35 [0.14–0.88], *P* = 0.019; stage 4, HR = 0.55 [0.38–0.79], *P* = 0.00094). However, no significant correlation was observed between the mRNA expression of *LPAR2* and OS in patients with HNSC tumor stage 1 (*P* > 0.05) (Fig. 9A–D). *LPAR2* overexpression was correlated with better OS in men with HNSC (HR = 0.58 [0.42–0.81], *P* = 0.0012); however, no significant correlation was found between *LPAR2* expression and OS in women with HNSC (*P* = 0.5) (Fig. 9E–F). For the criterion of race, higher mRNA expression of *LPAR2* was correlated with better OS in the White patients (HR = 0.64 [0.47–0.86], *P* = 0.0032) but not in the Black/Asian patients (*P* > 0.05) (Fig. 9G–H). Furthermore, we found that upregulated mRNA expression of *LPAR2* was associated with improved OS in patients with HNSC grade 2 (HR = 0.67 [0.47–0.96], *P* = 0.029) and grade 3 (HR = 0.33 [0.19–0.57], *P* = 3.5e-05) (Fig. 8J–K) but not in grade 1 (*P* > 0.05) (Fig. 9I). For the criterion of mutation status, the results indicated that high mRNA expression level of *LPAR2* were correlated with improved OS in the low-*LPAR2*-mutation-burden group (HR = 0.46 [0.29–0.74], *P* = 0.00095) (Fig. 9M). However, in the high-*LPAR2*-mutation-burden group, no significant relationship was observed between the mRNA expression of *LPAR2* and prognosis (*P* > 0.05) (Fig. 9L).

**Fig. 10:**
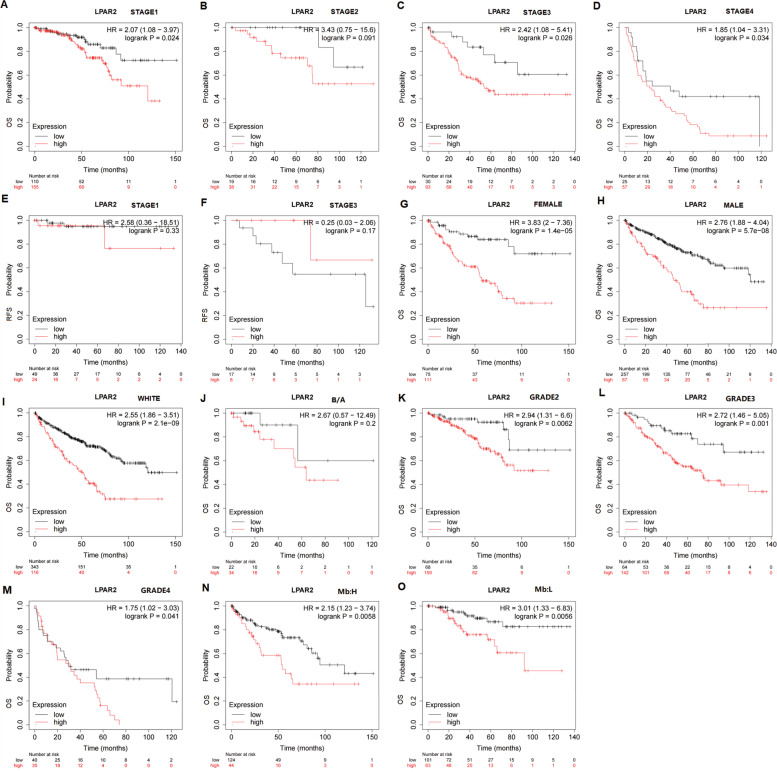
the relationship between the LPAR2 mRNA expression and prognosis in KIRC patients with different clinical characteristics in Kaplan–Meier plotter databases. Abbreviations: OS, overall survival; DFS, disease-free survival; RFS, relapse-free survival; DSS, disease-specific survival. DMFS, distant metastasis-free survival. Mb:H, Mutation burden high; Mb:L, Mutation burden low

In patients with KIRC, upregulated expression of *LPAR2* was associated with worse OS in patients with tumor stage 1 (HR = 2.07 [1.08–3.97], *P* = 0.024), tumor stage 3 (HR = 2.42 [1.08–5.41], *P* = 0.026), and tumor stage 4 (HR = 1.85 [1.04–3.31], *P* = 0.034) (Fig. 10A, C, D) but not in tumor stage 2 (*P* > 0.05) (Fig. 10B). In addition, high mRNA expression of *LPAR2* was associated with shorter OS in the White patients (HR = 2.55 [1.86–3.51], *P* = 2.1e-09) but not in the Black/Asian patients (*P* > 0.05) (Fig. 10I-J). *LPAR2* overexpression was associated with worse OS in men (HR = 2.76 [1.88–4.04], *P* = 5.7e-08) and women (HR = 3.83 [2–7.36], *P* = 1.4e-05) with KIRC (Fig. 10G–H). In addition, high *LPAR2* expression was associated with worse OS in patients with KIRC grade 2–4 (grade 2, HR = 2.94 [1.31–6.6], *P* = 0.0062; grade 3, HR = 2.72 [1.46–5.05], *P* = 0.001; and grade 4, HR = 1.75 [1.02–3.03], *P* = 0.041) (Fig. 10K–M) and in the high- and low-*LPAR2*-mutation-burden groups (high, HR = 2.15 [1.23–3.74], *P* = 0.0058; low, HR = 3.01 [1.33–6.83], *P* = 0.056) (Fig. 10N–O).

These results suggested that *LPAR2* expression influenced the prognosis of patients with HNSC of high stage and grade. Upregulated expression of *LPAR2* was beneficial to men with HNSC or patients with low *LPAR2* mutation burden and was significantly associated with prognosis in White patients with HNSC and KIRC.

### Association between *LPAR2* expression and immune cell infiltration in HNSC and KIRC

**Fig. 11:**
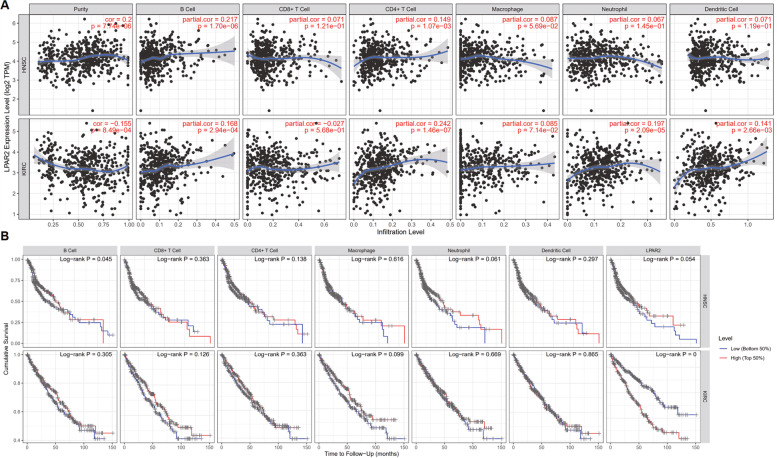
**A** Correlation of LPAR2 expression with immune infiltration level in HNSC and KIRC. **B** Kaplan–Meier plots of immune infiltration and LPAR2 expression levels in HNSC and KIRC

Tumor-infiltrating lymphocytes are independent predictors of tumor stage, grade, and lymph node status in cancers [25, 26]. Therefore, we used the TIMER database to analyze the relationship between *LPAR2* expression and the degree of immune cell infiltration in HNSC and KIRC (Fig. 11) and found that *LPAR2* expression was significantly correlated with tumor purity (*R* = 0.2, *P* = 7.74e-06), B cell infiltration (*R* = 0.217, *P* = 1.70e-05), and CD4 + T cell infiltration (*R* = 0.149, *P* = 1.07e-03) but not with the infiltration of CD8 + T cells, macrophages, neutrophils, and DCs in patients with HNSC (Fig. 11A). In patients with KIRC, *LPAR2* expression was significantly correlated with tumor purity (*R* = -0.155, *P* = 8.49e-04), B cell infiltration (*R* = 0.168, *P* = 2.94e-04), CD4 + T cell infiltration (*R* = 0.242 *P* = 1.46e-07), neutrophil infiltration (*R* = 0.197, *P* = 2.09e-05), and DC infiltration (*R* = 0.141, *P* = 2.66e-03) (Fig. 11A) but not with the infiltration of CD8 + T cells and macrophages (Fig. 11A). We further analyzed the correlation between *LPAR2* expressions and immune cell infiltration in patients with HNSC and KIRC by generating KM plots using the TIMER database. The results demonstrated that B-cell infiltration was significantly correlated with the prognosis of HNSC (*P* = 0.045) (Fig. 11B), and a significant correlation was observed between the mRNA expression of *LPAR2* and prognosis in patients with KIRC (*P* < 0.001) (Fig. 11B). These results suggest that *LPAR2* is important for regulating immune cell infiltration in HNSC and KIRC. Moreover, *LPAR2* is more important for regulating tumor purity and the infiltration of B cells and CD4 + T cells in HNSC as well as the infiltration of neutrophils and DCs in KIRC.

### Relationship between *LPAR2* and immune marker expression

Given that *LPAR2* is important for regulating immune cell infiltration in HNSC and KIRC, we assessed the relationship between *LPAR2* expression and immune cell infiltration based on the immunological markers of HNSC and KIRC using the TIMER and GEPIA databases. In addition, we evaluated the relationship between *LPAR2* expression and several immunological marker subsets, including total T cells, B cells, CD8 + T cells, tumor-associated macrophages (TAMs), monocytes, M1 and M2 macrophages, natural killer (NK) cells, neutrophils, DCs, T follicular helper (Tfh) cells, type 1 T helper (Th1) cells, Th2 cells, regulatory T cells (Tregs), Th17 cells, and exhausted T cells. All results were adjusted based on tumor purity. The results demonstrated a significant positive association between *LPAR2* expression and B cell markers (CD19 and CD79A), M1 macrophage markers (INOS and IRF5), neutrophil markers (CD11b), Th2 markers (*STAT6* and *STAT5A*), Tfh markers (*BCL6*), and T-cell exhaustion markers (CTLA4) in patients with HNSC (*P* < 0.01, Table 2). In patients with KIRC, a significant positive correlation was found between *LPAR2* expression and CD8 + T cell markers (CD8A and CD8B), total T cell markers (CD3D, CD3E, and CD2), B cell markers (CD19 and CD79A), monocyte markers (CD86 and CD115), TAM markers (CD68 and IL10), M1 macrophage markers (IRF5), M2 macrophage markers (CD163, VSIG4, and MS4A4A), neutrophil markers (CD11b and CCR7), NK cell markers (KIR2DL4), DC markers (HLA-DPB1, HLA-DRA, HLA-DPA1, and CD11C), Th1 markers (T-bet, STAT4, STAT1, IFN-γ, and TNF-α), Th2 markers (GATA3, STAT6, STAT5A, and IL13), Tfh markers (BCL6 and IL21), Treg markers (FOXP3, CCR8, STAT5B, and TGFβ), and T-cell exhaustion markers (PD-1, CTLA4, and LAG3) (*P* < 0.01, Table 2). However, *LPAR2* expression was negatively correlated with M1 macrophage markers (INOS), DC markers (BDCA-4), and Treg markers (STAT5B) in KIRC (Table 2).

**Table 2:** Correlation analysis between LPAR2 and relate genes and markers of immune cells in TIMER

**Description**	**Gene markers**	**HNSC**				**KIRC**			
		**None**		**Purity**		**None**		**Purity**	
		**Cor**	***P***	**Cor**	***P***	**Cor**	**P**	**Cor**	***P***
CD8 + T cell	CD8A	0.035	4.21e-01	0.101	2.52e-02	0.243	***	0.201	***
	CD8B	0.086	4.88e-02	0.144	*	0.26	***	0.226	***
T cell(general)	CD3D	0.102	2.04e-02	0.179	***	0.308	***	0.271	***
	CD3E	0.082	6.03e-02	0.159	**	0.303	***	0.263	***
	CD2	0.099	2.39e-02	0.166	**	0.289	***	0.244	***
B cell	CD19	0.185	***	0.256	***	0.354	***	0.309	***
	CD79A	0.157	**	0.216	***	0.308	***	0.265	***
Monocyte	CD86	0.009	8.45e-01	0.067	1.39e-01	0.232	***	0.215	***
	CD115(CSF1R)	0.024	5.91e-01	0.086	5.52e-02	0.289	***	0.266	***
TAM	CCL2	-0.019	6.65e-01	0.036	4.27e-01	-0.072	9.48e-02	-0.122	*
	CD68	-0.1	2.23e-02	-0.066	1.43e-01	0.227	***	0.241	***
	IL10	-0.063	1.50e-01	-0.002	9.66e-01	0.118	*	0.072	1.20e-01
M1Macrophage	INOS(NOS2)	0.262	***	0.252	***	-0.127	*	-0.127	*
	IRF5	0.173	***	0.18	***	0.301	***	0.301	***
	COX2(PTGS2)	-0.017	6.95e-01	-0.043	3.38e-01	0.057	1.92e-01	0.057	1.92e-01
M2 Macrophage	CD163	-0.017	6.92e-01	0.04	3.76e-01	0.123	*	0.113	1.53e-02
	VSIG4	-0.027	5.33e-01	0.034	4.52e-01	0.247	***	0.233	***
	MS4A4A	0.006	8.91e-01	0.069	1.28e-01	0.12	*	0.081	8.11e-02
Neutrophils	CD66b(CEACAM8)	0.053	2.29e-01	0.045	3.22e-01	-0.007	8.72e-01	-0.012	7.90e-01
	CD11b(ITGAM)	0.131	*	0.161	**	0.271	***	0.261	***
	CCR7	0.083	5.91e-02	0.154	**	0.275	***	0.25	***
Naturalkiller cell	KIR2DL1	0.026	5.51e-01	0.06	1.83e-01	-0.06	1.67e-01	-0.061	1.94e-01
	KIR2DL3	0.003	8.49e-01	0.043	3.46e-01	0.005	9.00e-01	0.024	6.13e-01
	KIR2DL4	0.009	8.44e-01	0.063	1.62e-01	0.136	*	0.122	*
	KIR3DL1	0.012	7.83e-01	0.042	3.57e-01	-0.066	1.29e-01	-0.051	2.73e-01
	KIR3DL2	0.036	4.01e-01	0.069	1.26e-01	0.059	1.71e-01	0.071	1.29e-01
	KIR3DL3	0.059	1.76e-01	0.094	3.78e-02	0.028	5.16e-01	0.012	8.01e-01
	KIR2DS4	0.052	2.38e-01	0.087	5.45e-02	0.004	9.36e-01	0.01	8.35e-01
**Dendritic cell**	HLA-DPB1	0.065	1.40e-01	0.135	2.64e-03	0.191	***	0.178	***
	HLA-DQB1	0.064	1.44e-01	0.108	1.63e-02	0.093	3.21e-02	0.073	1.19e-01
	HLA-DRA	0.014	7.52e-01	0.078	8.27e-02	0.128	*	0.111	1.67e-02
	HLA-DPA1	0.023	5.97e-01	0.085	5.92e-02	0.136	*	0.105	2.43e-02
	BCDA-1(CD1C)	0.019	6.66e-01	0.084	6.20e-02	0.04	3.58e-01	0.002	9.66e-01
	BDCA-4(NRP1)	-0.067	1.25e-01	-0.026	5.62e-01	-0.123	*	-0.169	**
	CD11c(ITGAX)	0.095	3.02e-02	0.173	*	0.315	***	0.313	***
Th1	T-bet (TBX21)	0.081	6.35e-02	0.142	*	0.145	**	0.112	1.62e-02
	STAT4	0.088	4.48e-02	0.145	*	0.297	***	0.259	***
	STAT1	-0.052	2.37e-01	-0.012	7.87e-01	0.195	***	0.154	**
	IFN-γ(IFNG)	0.017	7.05e-01	0.076	9.04e-02	0.284	***	0.243	***
	TNF-α(TNF)	0.052	2.32e-01	0.067	1.40e-01	0.24	***	0.212	***
Th2	GATA3	0.04	3.65e-01	0.078	8.50e-02	0.174	***	0.147	*
	STAT6	0.172	***	0.168	**	0.141	*	0.151	*
	STAT5A	0.23	***	0.255	9.09e-02	0.312	***	0.271	***
	IL13	0.075	8.69e-02	0.115	1.06e-02	0.153	**	0.126	*
Tfh	BCL6	0.264	***	0.236	***	0.292	***	0.279	***
	IL21	0.001	9.76e-01	0.03	5.06e-01	0.161	**	0.155	**
Th17	STAT3	0.056	2.01e-01	0.054	2.32e-01	0.029	4.99e-01	-0.003	9.41e-01
	IL17A	-0.013	7.69e-01	0.014	7.53e-01	0.058	1.79e-01	0.031	5.11e-01
Treg	FOXP3	0.08	6.9e-02	0.133	*	0.444	***	0.418	***
	CCR8	0.022	6.09e-01	0.062	1.71e-01	0.285	***	0.246	***
	STAT5B	0.084	5.61e-02	0.092	4.08e-02	-0.144	***	-0.156	**
	TGFβ(TGFB1)	-0.054	2.18e-01	-0.031	4.95e-01	0.29	***	0.265	***
T cell exhaustion	PD-1(PDCD1)	0.11	1.19e-02	0.174	**	0.378	***	0.357	***
	CTLA4	0.125	*	0.199	***	0.364	***	0.333	***
	LAG3	0.099	2.40e-02	0.152	**	0.359	***	0.321	***
	TIM-3(HAVCR2)	0.063	1.49e-01	0.123	*	-0.009	8.44e-01	-0.022	6.34e-01
	GZMB	0.039	3.76e-01	0.096	3.27e-02	0.103	1.71e-02	0.085	6.77e-02

Cor, *R* value of Spearman’s correlation; None, correlation without adjustment. Purity, correlation adjusted by purity. *P < 0 .01(1e-02); **P < 0.001(1e-03); ***P < 0.0001(1e-04)

*Abbreviations: HNSC* Head and Neck squamous cell carcinoma, *KIRC* kidney renal clear cell carcinoma, *TAM* tumor-correlated macrophage, *Tfh* follicular helper T cell, *Th* T helper cell, *Treg* regulatory T cell

The results suggested that *LPAR2* expression exhibited a significant correlation with the levels of most markers of B cells, M1 macrophages, Th2 cells, and Tfh cells in patients with HNSC (*P* < 0.0001, Table 2). Strikingly, in patients with HNSC, *LPAR2* expression was closely associated with INOS of M1 macrophages, *STAT5A* of Th2 cells, and *BCL6* of Tfh cells (*P* < 0.0001, Cor > 0.2, Table 2). In patients with KIRC, the mRNA expression of *LPAR2* was closely correlated with the levels of most markers of total CD8 + T cells (CD8A and CD8B), T cells (CD3D, CD3E, and CD2), B cells (CD19 and CD79A), monocytes (CD86 and CD115), TAMs (CD68), M1 macrophages (IRF5), M2 macrophages (VSIG4), neutrophils (CD11b and CCR7), DCs, Th1 cells (*STAT4*, IFN-γ, TNF-α), Th2 cells (*STAT5A*), Tfh cells (*BCL6*), Tregs (FOXP3, CCR8, and TGF-β), and exhausted T cells (PD-1, CTLA4, and LAG3) (*P* < 0.0001, Cor > 0.2, Table 2). Furthermore, we assessed the relationship between the expression of *LPAR2* and that of the aforementioned markers using GEPIA. The correlation between *LPAR2* expression and these markers was similar to that identified using TIMER (Table 3). These findings suggested that *LPAR2* was significantly correlated with infiltrating immune cells in HNSC and KIRC and played a significant role in the immune microenvironment of HNSC and KIRC.

**Table 3:** Correlation analysis between LPAR2 and relate genes and immune markers in GEPIA

**Description**	Gene markers	**HNSC**				**KIRC**				
		**Tumor**		**Normal**		**Tumor**			**Normal**	
		**R**	**P**	**R**	**P**	**R**		**P**	**R**	**P**
CD8 + T cell	CD8A	-0.012	0.79	0.059	0.7	0.26	***		0.027	0.82
	CD8B	0.028	0.52	0.06	0.7	0.27	***		-0.17	0.31
T cell(general)	CD3D	0.017	0.7	0.099	0.52	0.33	***		-0.003	0.8
	CD3E	0.029	0.51	0.13	0.41	0.32	***		-0.15	0.22
	CD2	0.038	0.13	0.4	0.33	0.3	***		-0.12	0.33
B cell	CD19	0.13	0.2	0.19	0.75	0.35	***		-0.15	0.2
	CD79A	0.093	*	0.34	*	0.3	***		-0.3	*
Monocyte	CD86	-0.017	0.7	0.23	0.14	0.24	***		-0.06	0.62
	CD115(CSF1R)	0.019	0.67	0.22	0.16	0.33	***		-0.072	0.55
TAM	CD68	-0.087	*	0.3	0.051	0.16	***		-0.06	0.62
M1Macrophage	INOS(NOS2)	0.25	***	0.19	0.22	-0.075	0.089		-0.001	0.68
	IRF5	0.16	***	0.57	***	0.35	***		-0.049	0.68
M2 Macrophage	VSIG4	-0.027	0.54	0.35	*	0.27	***		0.0054	0.96
Neutrophils	CD11b(ITGAM)	0.15	***	0.22	0.15	0.35	***		-0.16	0.17
	CCR7	0.054	0.22	0.14	0.36	0.28	***		-0.034	0.77
Dendritic cell	CD11c(ITGAX)	0.12	**	0.37	*	0.43	***		-0.12	0.33
Th1	STAT4	0.072	0.1	0.26	0.091	0.38	***		- 0.063	0.6
	IFN-γ(IFNG)	-0.025	0.56	-0.049	0.75	0.31	***		0.12	0.34
	TNF-α(TNF)	0.084	0.055	0.068	0.66	0.28	***		0.27	*
Th2	STAT5A	0.19	***	0.27	0.079	0.36	***		0.17	0.16
Tfh	BCL6	0.26	***	0.4	**	0.33	***		0.78	***
Treg	FOXP3	0.087	*	0.34	*	0.33	***		0.48	***
	CCR8	0.06	0.17	0.13	0.39	0.33	***		-0.16	0.17
	TGFβ(TGFB1)	0.039	0.38	0.39	**	0.34	***		0.76	***
Tcell exhaustion	PD-1(PDCD1)	0.051	0.25	0.099	0.52	0.4	***		-0.18	0.14
	CTLA4	0.086	0.051	0.16	0.31	0.42	***		-0.044	0.72
	LAG3	0.039	0.37	0.15	0.32	0.39	***		0.73	***

Cor, *R* value of Spearman’s correlation; * *P* < 0.05; ** *P* < 0.01; *** *P* < 0.001

*Abbreviations: HNSC* Head and Neck squamous cell carcinoma, *KIRC* kidney renal clear cell carcinoma, *TAM* Tumor-associated macrophages. Tumor, correlation analysis in tumor tissue of TCGA. Normal, correlation analysis in normal tissue of TCGA

### Alterations, mutations, methylations, and frequently altered neighbor genes of *LPAR2* in patients with HNSC and KIRC

**Fig. 12:**
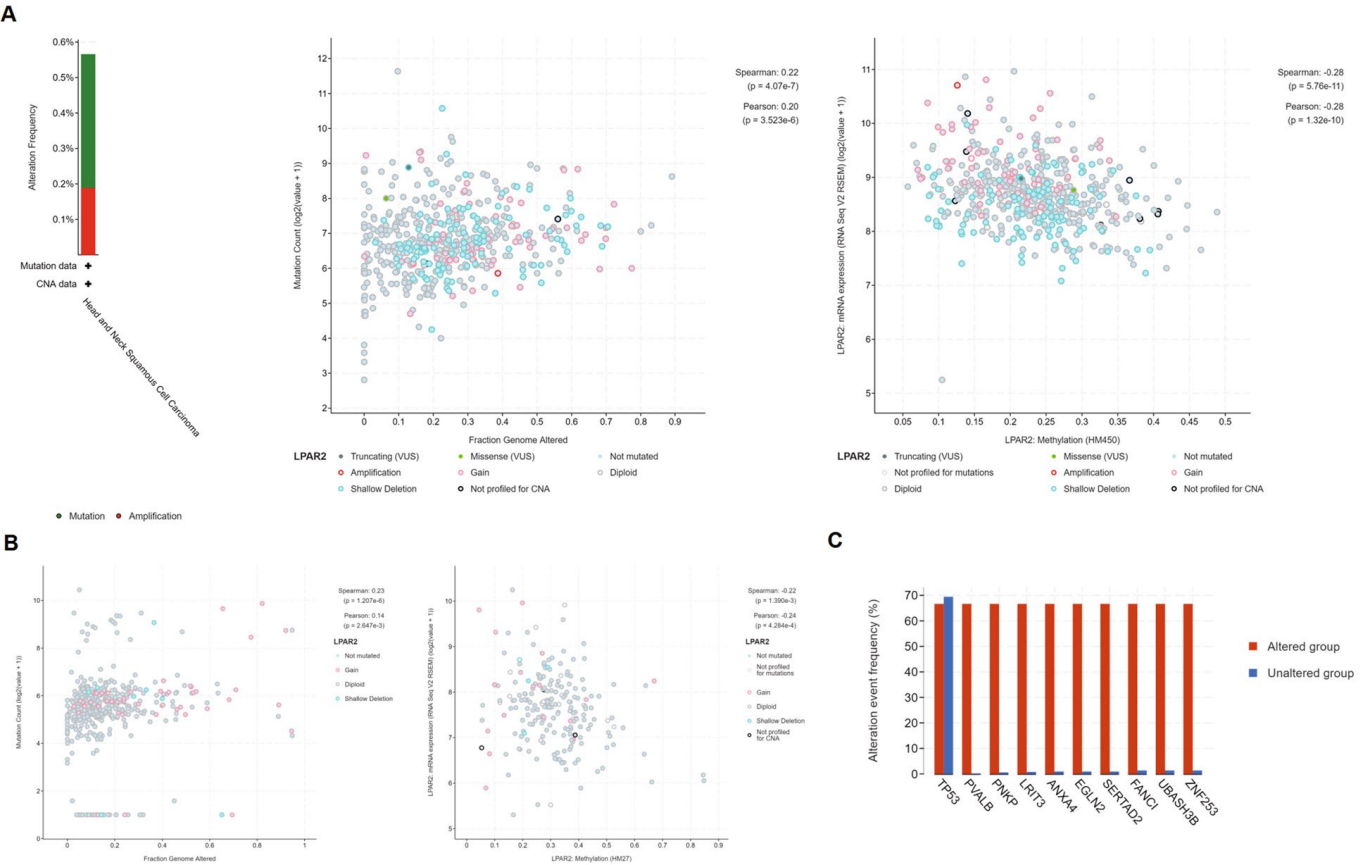
**A** Mutations and amplifications of LPAR2 in HNSC; **B** Alterations of LPAR2 in KIRC; **C** the 10 most frequently altered neighbor genes for LPAR2 in HNSC (cBioPortal)

We analyzed genetic alterations of *LPAR2* using the cBioPortal for Cancer Genomics in the HNSC and KIRC (TCGA, Firehose Legacy) datasets. *LPAR2* mutations and amplifications were found in 3 of 528 patients with HNSC but not in 537 patients with KIRC (Fig. 12A–B). In addition, we calculated the mutations, methylations, mRNA expression z-scores (RNA Seq V2 RSEM), protein expression Z-scores (RPPA), and putative CNAs of *LPAR2* in HNSC using GISTIC (Fig. 12A) and identified the 10 most frequently altered neighbor genes of *LPAR2* in HNSC (Fig. 12C). The results revealed that *LPAR2* alterations in HNSC were strongly associated with the mutated genes *TP53*, *PVALB*, *PNKP*, *LRIT3*, *ANXA4*, *EGLN2*, *SERTAD2*, *FANCI*, *UBASH3B*, and *ZNF253* (Fig. 12C).

## Discussion

LPA, a growth factor-like phospholipid, is abundantly found in human tissues and fluids [22]. It participates in various biological functions, such as cell migration, cell proliferation, inflammation, angiogenesis, and survival [27, 28]. LPA acts through G-protein-coupled LPA receptors, which are called LPARs [6, 8]. *LPAR2* belongs to the EDG family and contains 351 amino acids [22, 29]. It is unique in the proximal region of the C-terminus and contains several putative palmitoylated cysteine residues and a dileucine motif [30].

A few studies have suggested that *LPAR2* is associated with several cancers, such as breast [16, 31, 32], colon [20], ovarian [33], and stomach cancers [17]. These studies have reported that *LPAR2* expression is important in cancer biology and may promote gene transcription and cell proliferation in the tumor microenvironment [17, 34, 35]. However, the mechanism of action of *LPAR2* in tumors remains unclear.

In addition to traditional cancer treatment, cancer immunotherapy has emerged as an important therapy owing to its adequate efficacy and fewer side effects [36]. Nevertheless, immunotherapy has not been extensively investigated and effectively used to treat patients with HNSC and KIRC [37]. Given that immunotherapy mainly targets the tumor immune microenvironment, we analyzed the effects of *LPAR2* on tumor prognosis and immune infiltration of HNSC and KIRC in this study.

We examined the mRNA and protein expression levels of *LPAR2* in pan-cancer and the corresponding normal tissues using Oncomine, TIMER, UALCAN, and HPA databases, as well as validated by R software in TCGA and GEO databases. *LPAR2* expression was evaluated in tumor and normal tissues in multiple cancer types (Figs. 2 and 4, Table 1). Differences in data collection methods and analytical approaches may be attributed to the heterogeneity of *LPAR2* expression among cancer types and databases. However, we consistently observed higher expression of *LPAR2* in HNSC and KIRC across these databases.

We used online tools, such as KM plotter, GEPIA2.0, UACLAN and HPA, and R software to examine the critical role of *LPAR2* in predicting patient outcomes of multiple cancer types in TCGA and GEO databases. Our findings illustrated the expression levels and prognostic value of LPAR2 in several types of cancers, especially HNSC and KIRC (Figures S1, 2, 3, 4). High *LPAR2* expression was significantly correlated with a worse prognosis in KIRC. However, high *LPAR2* expression was strongly correlated with improved prognosis in HNSC. These contradictory results suggested that *LPAR2* acts as a tumor suppressor gene in HNSC and an oncogene in KIRC.

Given that *LPAR2* expression plays significantly different prognostic roles in HNSC and KIRC, we used UALCAN and KM plotter to examine the relationship between the mRNA expression of *LPAR2* and prognosis in patients with HNSC and KIRC with different clinical characteristics. The findings suggested that high *LPAR2* expression was associated with advanced tumor stages, high tumor grades, and lymph node metastasis in patients with KIRC. Using KM plotter, we found that high *LPAR2* expression was associated with improved prognosis in patients with HNSC with advanced tumor stages and high tumor grades. Meanwhile, high *LPAR2* expression resulted in better prognosis in patients with HNSC, which may be related to their mutational burden status. These results means that *LPAR2* was involved in tumor development and progression of patients with HNSC or KIRC.

Given that high *LPAR2* expression affects prognosis related to clinical characteristics in HNSC and KIRC patients, we assessed the relationship between *LPAR2* expression and the degree of immune cell infiltration using the TIMER database. Another important finding of this study was that *LPAR2* expression was significantly associated with the infiltration of diverse immune cells in HNSC and KIRC. We found that *LPAR2* expression had a positive correlation with tumor purity in HNSC and KIRC, the infiltration of B cells and CD4 + T cells in HNSC (Fig. 11A), and the infiltration of B cells, CD4 + T cells, neutrophils, and DCs in KIRC (Fig. 11A). These results suggest that *LPAR2* is important for regulating immune cell infiltration in HNSC and KIRC, with particularly strong effects on tumor purity and infiltrating B cells, CD4 + T cells, neutrophils, and DCs.

Furthermore, to investigate the role of *LPAR2* in the regulation of tumor immunology in HNSC and KIRC, we analyzed the relationship between *LPAR2* expression and marker genes of immune cells. We found a significant positive correlation between *LPAR2* expression and the markers of B cells (CD19 and CD79A), M1 macrophages (INOS and IRF5), neutrophils (CD11b), Th2 cells (*STAT6* and *STAT5A*), Tfh cells (*BCL6*), and exhausted T cells (CTLA4) in HNSC (*P* < 0.01, Table 2). In addition, *LPAR2* expression was strongly correlated with INOS of M1 macrophages, *STAT5A* of Th2 cells, and *BCL6* of Tfh cells (*P* < 0.0001, Cor > 0.2, Table 2). These results indicate that *LPAR2* promotes the polarization of macrophages to the M1 phenotype and regulates T cell responses. Furthermore, *BCL6* recognizes DNA target sequences similar to those recognized by *STAT5 *[38]. Some studies have found that *STAT5A* inhibits cell invasion and metastasis in breast cancer [39]. *LPAR2* may play a role in HNSC by interacting with STAT5A and BCL6 via the prolactin–JAK2–STAT5A pathway [38]; but further studies are warranted. In this study, *LPAR2* expression was significantly correlated with most immune markers in KIRC, including CD3D and CD3E of total T cells; CD19 and CD79A of B cells; IRF5 of M1 macrophages; *STAT5A* of Th2 cells; FOXP3 and CCR8 of Treg cells; and PD-1, CTLA4, and LAG3 of exhausted T cells (*P* < 0.0001, Cor > 0.3, Table 2). In addition, the results indicate that *LPAR2* activates Tregs and B cells, induces T cell exhaustion, and promotes Treg responses to suppress T cell-mediated immunity, thereby regulating T cell responses in KIRC. *LPAR2* may promote the polarization of macrophages to the M1 phenotype via IRF5. Therefore, these findings collectively suggest that *LPAR2* is a crucial factor for the recruitment and regulation of infiltrating immune cells in HNSC and KIRC.

## Conclusion

*LPAR2* plays significantly different prognostic roles in HNSC and KIRC might owing to its association with different immune markers. *LPAR2* is important for governing immune cell infiltration, and is a valuable prognostic biomarker that may guide treatment in HNSC and KIRC. Nevertheless, further validation experiments are required.

## Materials and methods

### Data processing and differential expression analysis, survival analysis and correlation analysis

The UCSC Xena dataset was used to acquire TCGA expression and clinical information (https://toil-xena-hub.s3.us-east-1.amazonaws.com/download/TcgaTargetGtex_rsem_gene_tpm.gz; Full metadata) [40]. Dataset ID: TcgaTargetGtex_rsem_gene_tpm. Raw counts of RNA-sequencing data (level 3) and matching clinical data contains 10,363 tumor tissues and 730 adjacent tissues from 18 types of cancer. Eight independent HNSC and KIRC/RCC gene expression profiles (GSE30784, GSE31056, GSE686, GSE65858, GSE53757, GSE15641, GSE167573 and GSE22541) were downloaded from the Gene Expression Omnibus (GEO) database (https://www.ncbi.nlm.nih.gov/geo/)  [41] and processed for analysis. Detailed information of datasets was listed in Table 4. All analytical methods were carried out utilizing the R software version v4.0.3. Expression analysis and Survival curves were drawn using the R packages “ggplot2”, “survival”, and “survminer”[42, 43]. The Log-rank tests as well as the univariate Cox proportional hazards regression generated hazard ratio (HR) and p-values with a confidence interval (CI) of 95% in KM curves.

**Table 4:** Information of the Selected GEO Datasets

Datasets	Contributor	Disease type	Experimental platform	Number of cases (cancer/control)
GSE30784	Chen C, et al. (2011)	OSCC	Affymetrix Human Genome U133 Plus 2.0 Array	167/62
GSE31056	Reis PP, et al.(2011)	OSCC	Affymetrix GeneChip Human Genome HG-U133 Plus 2 Array	23/24
GSE686	Chung CH, et al. (2004)	HNSC	Agilent Human 1 cDNA Microarray	78/0
GSE65858	Wichmann G, et al.(2015)	HNSC	Illumina HumanHT-12 V4.0 expression beadchip	270/0
GSE53757	von Roemeling CA, et al.(2014)	KIRC	Affymetrix Human Genome U133 Plus 2.0 Array	72/72
GSE15641	Jones J, et al. (2005)	RCC	Affymetrix Human Genome U133A Array	69/23
GSE167573	He N, et al.(2021)	RCC	HiSeq X Ten (Homo sapiens)	63/14
GSE22541	Wuttig D, et al.(2012)	KIRC	Affymetrix Human Genome U133 Plus 2.0 Array	68/0

*Abbreviations: OSCC* oral squamous cell carcinoma, *HNSC* Head and Neck squamous cell carcinoma, *KIRC* kidney renal clear cell carcinoma, *RCC* renal cell cancer

### Oncomine database analysis

The expression data of 715 genes were obtained from 86,733 samples and the mRNA expression levels of *LPAR2* in pan-cancer were analyzed using the online cancer microarray database (Oncomine) (www.oncomine.org). The Student’s t-test was used to compare the mRNA expression of *LPAR2* between normal and cancer samples. *P*-value was used to characterize significant differences. The fold change was 1.5, and the cut-off *P-*value was 0.0001.

### TIMER database analysis

The Tumor Immune Estimation Resource (TIMER) (https://cistrome.shinyapps.io/timer/) database comprises six tumor-infiltrating immune cell subsets [44], and the expression levels of six subsets are pre-calculated for 10,897 tumors across 32 cancer types from The Cancer Genome Atlas (TCGA). The database allows the analysis of gene expression and tumor immune infiltration (B cells, CD4 + T cells, CD8 + T cells, neutrophils, macrophages, and dendritic cells [DCs]) in various cancer types. In this study, TIMER was used to analyze the mRNA expression of *LPAR2* in various cancer types and investigate the relationship between *LPAR2* expression and the degree of infiltration of specific immune cell subsets. Furthermore, differences in the survival of patients with cancer based on gene expression or immune cell infiltration were examined using KM survival analysis. Lastly, the correlation between the expression of *LPAR2* and that of specific immune markers was examined.

### UALCAN

UALCAN (http://ualcan.path.uab.edu/index.html) is an interactive web resource used for analyzing publicly available cancer omics data(TCGA, MET500, and Clinical Proteomic Tumor Analysis Consortium) [45]. In this study, UALCAN was used to examine the mRNA expression level of *LPAR2* in different cancer and normal samples using the TCGA data and investigate the relationship between *LPAR2* expression and different clinical characteristics. In addition, the prognostic value of *LPAR2* in pan-cancer and the relationship between *LPAR2* expression and the prognosis of patients with different clinical characteristics were analysed.

### KM plotter analysis

The KM plotter (http://kmplot.com/analysis/) is an online database, which contains microarray gene expression data and survival information derived from the European Genome-Phenome Archive, Gene Expression Omnibus (GEO), and TCGA. It is used to assess the influence of multiple genes on the survival rate in 21 cancer types in a large number of samples [46]. In this study, the KM plotter was used to analyze the prognostic value of *LPAR2* in pan-cancer and investigate the relationship between *LPAR2* expression and the prognosis of patients with different clinical characteristics.

### GEPIA2 database analysis

GEPIA (http://gepia.cancer-pku.cn/index.html) uses standard processing pipelines to analyze the RNA-sequencing expression data of 8,587 normal samples and 9,736 tumors from the GTEx and TCGA projects [47]. GEPIA2 (http://gepia2.cancer-pku.cn/#index) is an updated version of GEPIA [48]. In this study, GEPIA2 was used to examine the relationship between the mRNA expression of *LPAR2* and pan-cancer prognosis as well as the relationship between the expression of *LPAR2* and that of the markers of immune cell infiltration.

### HPA database

The Human Protein Atlas (HPA) database (www.proteinatlas.org) was used to analyze the protein expression of *LPAR2* in HNSC, KIRC, and normal tissues [49, 50]. HPA provides access to the protein expression profiles of 32 human tissues and uses antibody profiling to accurately assess protein localization. In addition, it provides the measurements of RNA levels. In this study, HPA was used to visualize the representative immunohistochemical images of *LPAR2* in HNSC, KIRC, and their corresponding normal tissues. In addition, the relationship between the protein expression level of *LPAR2* and the prognosis of patients with HNSC and KIRC was examined.

### TCGA and cBioPortal for cancer genomics

The cBioPortal for Cancer Genomics tool (http://www.cbioportal.org) is used to analyze, visualize, and download cancer genomics datasets [51]. In this study, the cBioPortal for Cancer Genomics was used to download the HNSC and KIRC (TCGA, Firehose Legacy) datasets for *LPAR2* analysis, which contained histopathological data of 528 patients with HNSC and 537 patients with KIRC. The genomic profiles were evaluated via the Genomic Identification of Significant Targets in Cancer (GISTIC) analysis and included the assessment of mutations, methylations, mRNA expression z-scores (RNA Seq V2 RSEM), protein expression z-scores (RPPA), and putative copy number alterations (CNAs). Co-expression was evaluated according to the instructions provided on cBioPortal.

### Statistical analysis

Data were analyzed using the log-rank test, which included fold change, hazard ratio (HR), and *P-*values. Furthermore, the degree of relationship between specific variables was measured via Spearman’s correlation analysis, with R values, to measure the relationship strength as follows: “very weak”, 0.00–0.19; “weak”, 0.20–0.39; “moderate”, 0.40–0.59; “strong”, 0.60–0.79; and “very strong”, 0.80–1.0. A *P*-value < 0.05 indicated statistical significance.

## Supplementary Information

**Addtional file 1: Table S1.** Clinical characteristics of patients in HPA.

**Addtional file 2: Table S2.** Clinical characteristics of patients with HNSC in HPA.

**Addtional file 3: Table S3.** Clinical characteristics of patients with KIRC in HPA.

**Addtional file 4: Figure S1.** Kaplan-Meier survival curves comparing the high and low expression of LPAR2 in different types of cancers in the Kaplan-Meier plotter databases(A-AH).

**Addtional file 5: Figure S2.** Kaplan-Meier survival curves comparing the high and low expression of LPAR2 in different types of cancer in GEPIA databases(A-BB).

**Addtional file 6: Figure S3.** Kaplan-Meier survival curves comparing the high and low expression of LPAR2 in different types of cancer in UACLAN databases(A-S).

**Addtional file 7: Figure S4.** Kaplan-Meier survival curves comparing the high and low expression of LPAR2 in different types of cancer in TCGA databases(A-AG).

**Addtional file 8: Figure S5.** Kaplan-Meier survival curves comparing the high and low expression of LPAR2 in HNSC and KIRC from GEO databases and the paired ROC curves of measuring the predictive value(A-D).

## Data Availability

All the datasets were retrieved from the publishing literature, so it was confirmed that all written informed consent was obtained.

## Abbreviations

LPAR2: Lysophosphatidic acid receptors 2
TIMER: Tumor Immune Estimation Resource
HPA: Human Protein Atlas
GEPIA: Gene Expression Profiling Interactive Analysis
TCGA: The Cancer Genome Atlas
GTEx: The Genotype-Tissue Expression
ONCOMINE: Online cancer microarray database
K-M plotter: Kaplan–Meier plotter
HNSC: Head and neck squamous cell carcinoma
KIRC: Kidney renal clear cell carcinoma
ATX: Autotaxin
BC: Breast cancer
RNA Seq V2 RSEM: MRNA expression z-scores
RPPA: Protein expression Z-scores
CNA: Copy-number alterations
ECL: Extracellular loop
EDG: Endothelial differentiation gene
GEO: Gene Expression Omnibus
GEPIA: Gene Expression Profiling Interactive Analysis
GPCRs: G-protein coupled receptors
HR: Hazard ratio
LP: Lysophospholipid
LPA: Lysophosphatidic acid
LPARs: Lysophosphatidic acid receptors
BLCA: Bladder urothelial carcinoma
BRCA: Breast invasive carcinoma
KICH: Kidney chromophobe
KIRP: Kidney renal papillary cell carcinoma
LIHC: Liver hepatocellular carcinoma
ESCA: Esophageal carcinoma
LUAD: Lung adenocarcinoma
LUSC: Lung squamous cell carcinoma
PRAD: Prostate adenocarcinoma
READ: Rectum adenocarcinoma
UCEC: Uterine corpus endometrial carcinoma
CHOL: Cholangial carcinoma
COAD: Colon adenocarcinoma
CECS: Cervical squamous cell carcinoma and endocervical adenocarcinoma
STAD: Stomach adenocarcinoma
DLBC: Lymphoid Neoplasm Diffuse Large B-cell Lymphoma
THCA: Thyroid carcinoma
GBM: Glioblastoma multiforme
PAAD: Pancreatic adenocarcinoma
THYM: Thymoma
ACC: Adrenocortical carcinoma
BLCA: Bladder urothelial carcinoma
PCPG: Pheochromocytoma and Paraganglioma
UCEC: Uterine corpus endometrial carcinoma
UCS: Uterine Carcinosarcoma
OS: Overall survival
DFS: Disease-free survival
RFS: Relapse-free survival
PPS: Post-progression survival
DSS: Disease-specific survival
DMFS: Distant metastasis-free survival
FP: First progression
TAM: Tumor-associated macrophages
NK cells: Natural killer cells
DCs: Dendritic cells
Tfh: Follicular helper T cell
Th: T helper cell
HR: Hazard ratio
OSCC: Oral squamous cell carcinoma
RCC: Renal cell cancer

## References

[1] Liu S, Umezu-Goto M, Murph M (2009). Expression of autotaxin and lysophosphatidic acid receptors increases mammary tumorigenesis, invasion, and metastases. Cancer Cell.

[2] Hu HB, Song ZQ, Song GP (2021). LPA signaling acts as a cell-extrinsic mechanism to initiate cilia disassembly and promote neurogenesis. Nat Commun.

[3] Network CGA (2012). Comprehensive molecular portraits of human breast tumours. Nature.

[4] Hartman ZC, Poage GM, den Hollander P (2013). Growth of triple-negative breast cancer cells relies upon coordinate autocrine expression of the proinflammatory cytokines IL-6 and IL-8. Cancer Res.

[5] Chrencik JE, Roth CB, Terakado M (2015). Crystal Structure of Antagonist Bound Human Lysophosphatidic Acid Receptor 1. Cell.

[6] Tager AM, LaCamera P, Shea BS (2008). The lysophosphatidic acid receptor LPA1 links pulmonary fibrosis to lung injury by mediating fibroblast recruitment and vascular leak. Nat Med.

[7] Farquhar MJ, Humphreys IS, Rudge SA (2017). Autotaxin-lysophosphatidic acid receptor signalling regulates hepatitis C virus replication. J Hepatol.

[8] Llona-Minguez S, Ghassemian A, Helleday T (2015). Lysophosphatidic acid receptor (LPAR) modulators: The current pharmacological toolbox. Prog Lipid Res.

[9] Marshall JC, Collins JW, Nakayama J (2012). Effect of inhibition of the lysophosphatidic acid receptor 1 on metastasis and metastatic dormancy in breast cancer. J Natl Cancer Inst.

[10] Hama K, Aoki J (2010). LPA(3), a unique G protein-coupled receptor for lysophosphatidic acid. Prog Lipid Res.

[11] Mansell JP, Barbour M, Moore C (2010). The synergistic effects of lysophosphatidic acid receptor agonists and calcitriol on MG63 osteoblast maturation at titanium and hydroxyapatite surfaces. Biomaterials.

[12] Mazzocca A, Dituri F, De Santis F (2015). Lysophosphatidic acid receptor LPAR6 supports the tumorigenicity of hepatocellular carcinoma. Cancer Res.

[13] Zhang H, Xu X, Gajewiak J (2009). Dual activity lysophosphatidic acid receptor pan-antagonist/autotaxin inhibitor reduces breast cancer cell migration in vitro and causes tumor regression in vivo. Cancer Res.

[14] Allanore Y, Distler O, Jagerschmidt A (2018). Lysophosphatidic Acid Receptor 1 Antagonist SAR100842 for Patients With Diffuse Cutaneous Systemic Sclerosis: A Double-Blind, Randomized, Eight-Week Placebo-Controlled Study Followed by a Sixteen-Week Open-Label Extension Study. Arthritis Rheumatol.

[15] Szepanowski F, Winkelhausen M, Steubing RD, Mausberg AK, Kleinschnitz C, Stettner M (2021). LPA(1) signaling drives Schwann cell dedifferentiation in experimental autoimmune neuritis. J Neuroinflammation.

[16] Sun K, Cai H, Duan X (2015). Aberrant expression and potential therapeutic target of lysophosphatidic acid receptor 3 in triple-negative breast cancers. Clin Exp Med.

[17] Ren Z, Zhang C, Ma L (2019). Lysophosphatidic acid induces the migration and invasion of SGC-7901 gastric cancer cells through the LPA2 and Notch signaling pathways. Int J Mol Med.

[18] Takahashi K, Fukushima K, Tanaka K (2018). Involvement of LPA signaling via LPA receptor-2 in the promotion of malignant properties in osteosarcoma cells. Exp Cell Res.

[19] Park J, Jang JH, Oh S (2018). LPA-induced migration of ovarian cancer cells requires activation of ERM proteins via LPA(1) and LPA(2). Cell Signal.

[20] Shukla PK, Meena AS, Gangwar R (2020). LPAR2 receptor activation attenuates radiation-induced disruption of apical junctional complexes and mucosal barrier dysfunction in mouse colon. FASEB J.

[21] Kuriyama S, Theveneau E, Benedetto A (2014). In vivo collective cell migration requires an LPAR2-dependent increase in tissue fluidity. J Cell Biol.

[22] Deng W, Shuyu E, Tsukahara R (2007). The lysophosphatidic acid type 2 receptor is required for protection against radiation-induced intestinal injury. Gastroenterology.

[23] Chen M, Towers LN, O'Connor KL (2007). LPA2 (EDG4) mediates Rho-dependent chemotaxis with lower efficacy than LPA1 (EDG2) in breast carcinoma cells. Am J Physiol Cell Physiol.

[24] Spaniol B, Lang J, Venn B (2022). Complexome profiling on the Chlamydomonas lpa2 mutant reveals insights into PSII biogenesis and new PSII associated proteins. J Exp Bot.

[25] Azimi F, Scolyer RA, Rumcheva P (2012). Tumor-infiltrating lymphocyte grade is an independent predictor of sentinel lymph node status and survival in patients with cutaneous melanoma. J Clin Oncol.

[26] Ohtani H (2007). Focus on TILs: prognostic significance of tumor infiltrating lymphocytes in human colorectal cancer. Cancer Immun.

[27] Taniguchi R, Inoue A, Sayama M (2017). Structural insights into ligand recognition by the lysophosphatidic acid receptor LPA6. Nature.

[28] Gento-Caro Á, Vilches-Herrando E, García-Morales V (2021). Interfering with lysophosphatidic acid receptor edg2/lpa(1) signalling slows down disease progression in SOD1-G93A transgenic mice. Neuropathol Appl Neurobiol.

[29] Lin S, Wang D, Iyer S (2009). The absence of LPA2 attenuates tumor formation in an experimental model of colitis-associated cancer. Gastroenterology.

[30] Lee SJ, Ritter SL, Zhang H, Shim H, Hall RA, Yun CC (2011). MAGI-3 competes with NHERF-2 to negatively regulate LPA2 receptor signaling in colon cancer cells. Gastroenterology.

[31] Kitayama J, Shida D, Sako A (2004). Over-expression of lysophosphatidic acid receptor-2 in human invasive ductal carcinoma. Breast Cancer Res.

[32] Sun K, Duan X, Cai H (2016). Curcumin inhibits LPA-induced invasion by attenuating RhoA/ROCK/MMPs pathway in MCF7 breast cancer cells. Clin Exp Med.

[33] Kowalczyk-Zieba I, Woclawek-Potocka I, Wasniewski T (2019). LPAR2 and LPAR4 are the Main Receptors Responsible for LPA Actions in Ovarian Endometriotic Cysts. Reprod Sci.

[34] Hasse S, Duchez AC, Fortin P, Boilard E, Bourgoin SG (2021). Interplay between LPA2 and LPA3 in LPA-mediated phosphatidylserine cell surface exposure and extracellular vesicles release by erythrocytes. Biochem Pharmacol..

[35] Cecchin M, Jeong J, Son W (2021). LPA2 protein is involved in photosystem II assembly in Chlamydomonas reinhardtii. Plant J.

[36] Tang Y, Xu Q, Hu L, et al. Tumor Microenvironment-Derived R-spondins Enhance Anti-Tumor Immunity to Suppress Tumor Growth and Sensitize for Immune Checkpoint Blockade Therapy. Cancer Discov. 2021;candisc.0833.2020. Online ahead of print. 10.1158/2159-8290.CD-20-0833 PMC8716674 34193438

[37] Sung H, Ferlay J, Siegel RL (2021). Global Cancer Statistics 2020: GLOBOCAN Estimates of Incidence and Mortality Worldwide for 36 Cancers in 185 Countries. CA Cancer J Clin.

[38] Tran TH, Utama FE, Lin J (2010). Prolactin inhibits BCL6 expression in breast cancer through a Stat5a-dependent mechanism. Cancer Res.

[39] Tran TH, Utama FE, Sato T (2018). Loss of Nuclear Localized Parathyroid Hormone-Related Protein in Primary Breast Cancer Predicts Poor Clinical Outcome and Correlates with Suppressed Stat5 Signaling. Clin Cancer Res.

[40] Goldman MJ, Craft B, Hastie M (2020). Visualizing and interpreting cancer genomics data via the Xena platform. Nat Biotechnol.

[41] Barrett T, Wilhite SE, Ledoux P (2013). NCBI GEO: archive for functional genomics data sets–update. Nucleic Acids Res..

[42] Ito K, Murphy D (2013). Application of ggplot2 to Pharmacometric Graphics. CPT Pharmacometrics Syst Pharmacol..

[43] Yu G, Wang LG, Han Y, He QY (2012). clusterProfiler: an R package for comparing biological themes among gene clusters. OMICS.

[44] Li T, Fan J, Wang B (2017). TIMER: A Web Server for Comprehensive Analysis of Tumor-Infiltrating Immune Cells. Cancer Res.

[45] Chandrashekar DS, Bashel B, Balasubramanya S (2017). UALCAN: A Portal for Facilitating Tumor Subgroup Gene Expression and Survival Analyses. Neoplasia.

[46] Nagy Á, Munkácsy G, Győrffy B (2021). Pancancer survival analysis of cancer hallmark genes. Sci Rep.

[47] Tang Z, Li C, Kang B, Gao G, Li C, Zhang Z (2017). GEPIA: a web server for cancer and normal gene expression profiling and interactive analyses. Nucleic Acids Res.

[48] Tang Z, Kang B, Li C, Chen T, Zhang Z (2019). GEPIA2: an enhanced web server for large-scale expression profiling and interactive analysis. Nucleic Acids Res.

[49] Uhlén M, Fagerberg L, Hallström BM (2015). Proteomics. tissue-based map of the human proteome. Science..

[50] Karlsson M, Zhang C, Méar L, et al. A single-cell type transcriptomics map of human tissues. Sci Adv. 2021;7(31):eabh2169. 10.1126/sciadv.abh2169 PMC8318366 34321199

[51] Gao J, Aksoy BA, Dogrusoz U (2013). Integrative analysis of complex cancer genomics and clinical profiles using the cBioPortal. Sci Signal..

