# Supplementary material for: LPAR2 correlated with different prognosis and immune cell infiltration in head and neck squamous cell carcinoma and kidney renal clear cell carcinoma
Source: Hereditas. 2022 Mar 4;159:16. doi: 10.1186/s41065-022-00229-w (PMC8896370; doi:10.1186/s41065-022-00229-w)
Supplement: Supplementary file 1 — Addtional file 1: Table S1. Clinical characteristics of patients in HPA. [file 41065_2022_229_MOESM1_ESM.docx]

Table S1 Clinical characteristics of patients in HPA

| Number | Sample | Description | Staining |
| --- | --- | --- | --- |
| Figure5A | Oral Normal tissue | Male, age 66  Squamous epithelial cells | Not detected |
| Figure5B | Oral Squamous cell carcinoma tissue | Female, age 50  Squamous cell carcinoma cells | Medium |
| Figure5C | Kidney normal tissue | Female, age 56  Cells in glomeruli | Not detected |
| Figure5D | Kidney Adenocarcinoma | Male, age 63  Kidney Adenocarcinoma cells | Medium |
