# Supplementary figures and images for: LPAR2 correlated with different prognosis and immune cell infiltration in head and neck squamous cell carcinoma and kidney renal clear cell carcinoma
Source: Hereditas. 2022 Mar 4;159:16. doi: 10.1186/s41065-022-00229-w (PMC8896370; doi:10.1186/s41065-022-00229-w)

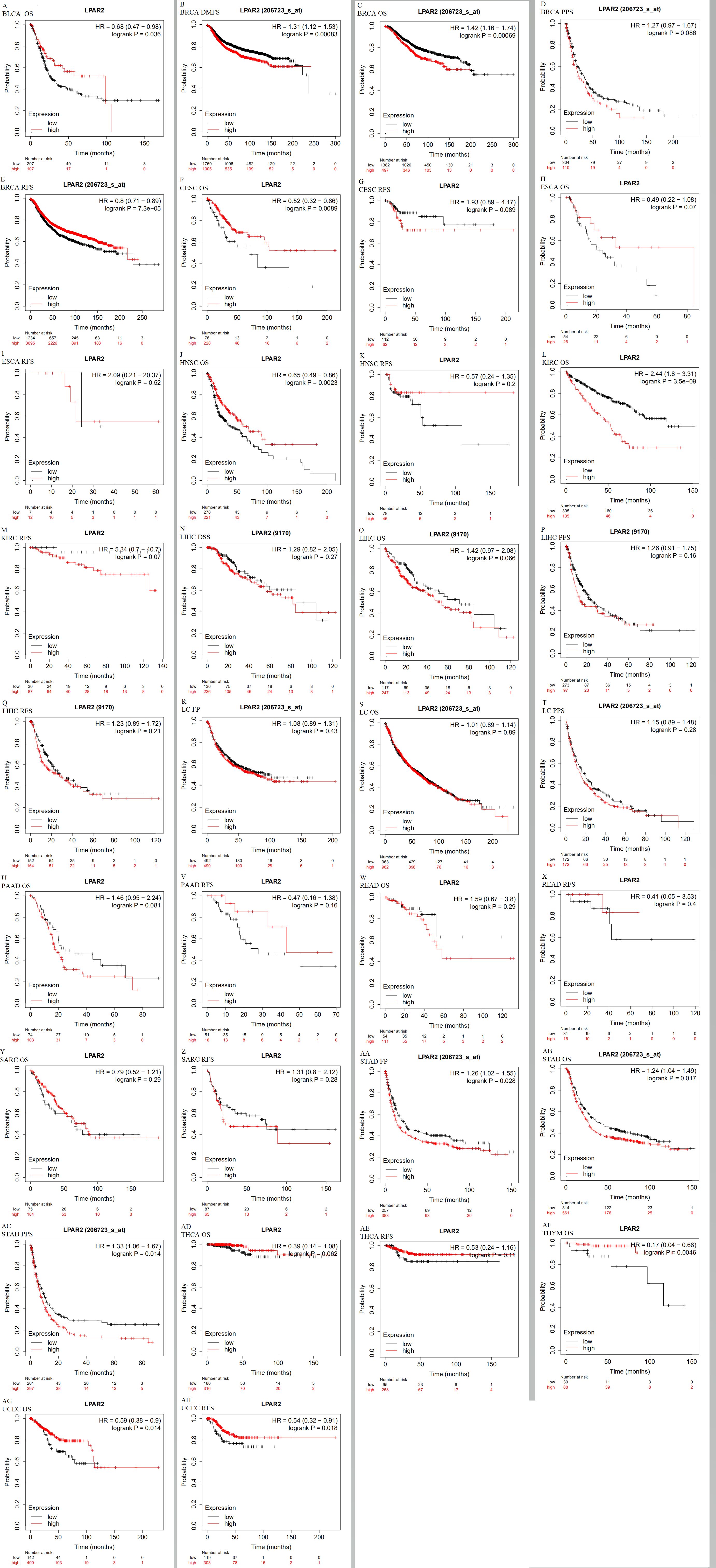

Supplement: Supplementary file 4 — Addtional file 4: Figure S1. Kaplan-Meier survival curves comparing the high and low expression of LPAR2 in different types of cancers in the Kaplan-Meier plotter databases(A-AH). [file 41065_2022_229_MOESM4_ESM.tiff]

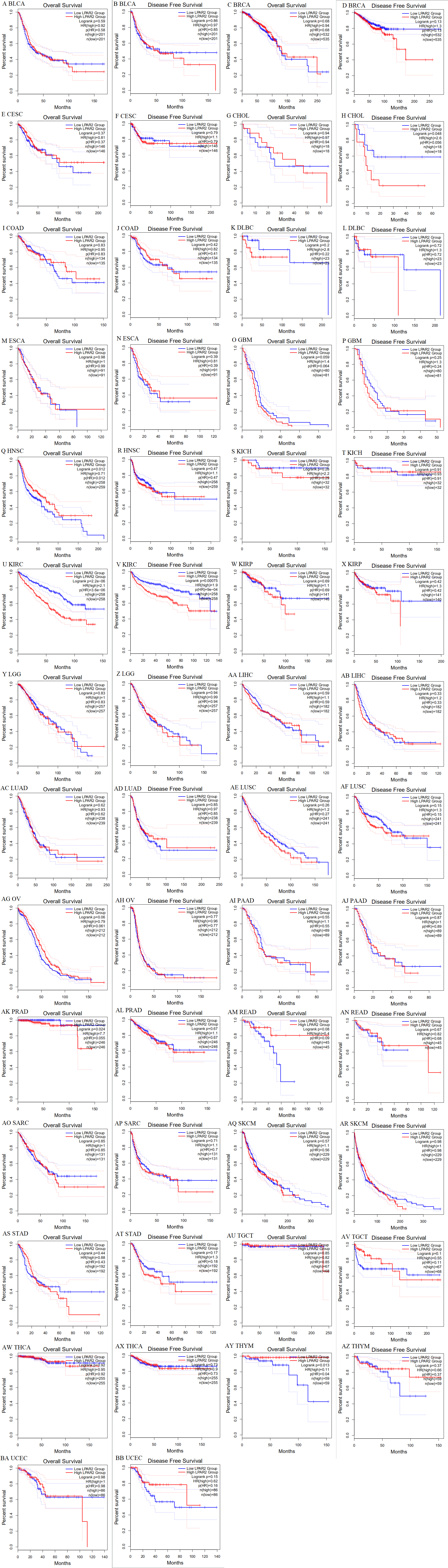

Supplement: Supplementary file 5 — Addtional file 5: Figure S2. Kaplan-Meier survival curves comparing the high and low expression of LPAR2 in different types of cancer in GEPIA databases(A-BB). [file 41065_2022_229_MOESM5_ESM.tiff]

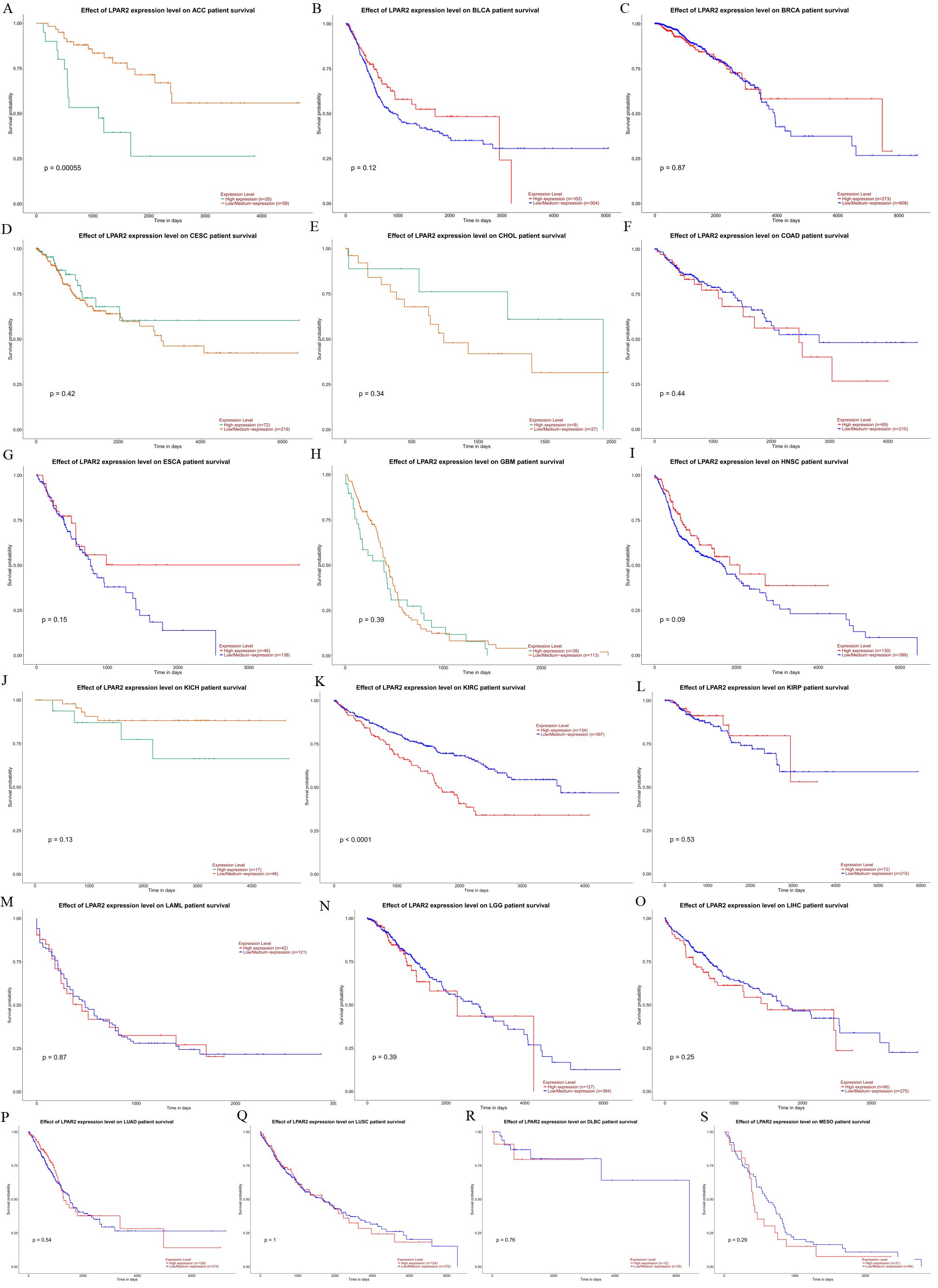

Supplement: Supplementary file 6 — Addtional file 6: Figure S3. Kaplan-Meier survival curves comparing the high and low expression of LPAR2 in different types of cancer in UACLAN databases(A-S). [file 41065_2022_229_MOESM6_ESM.tif]

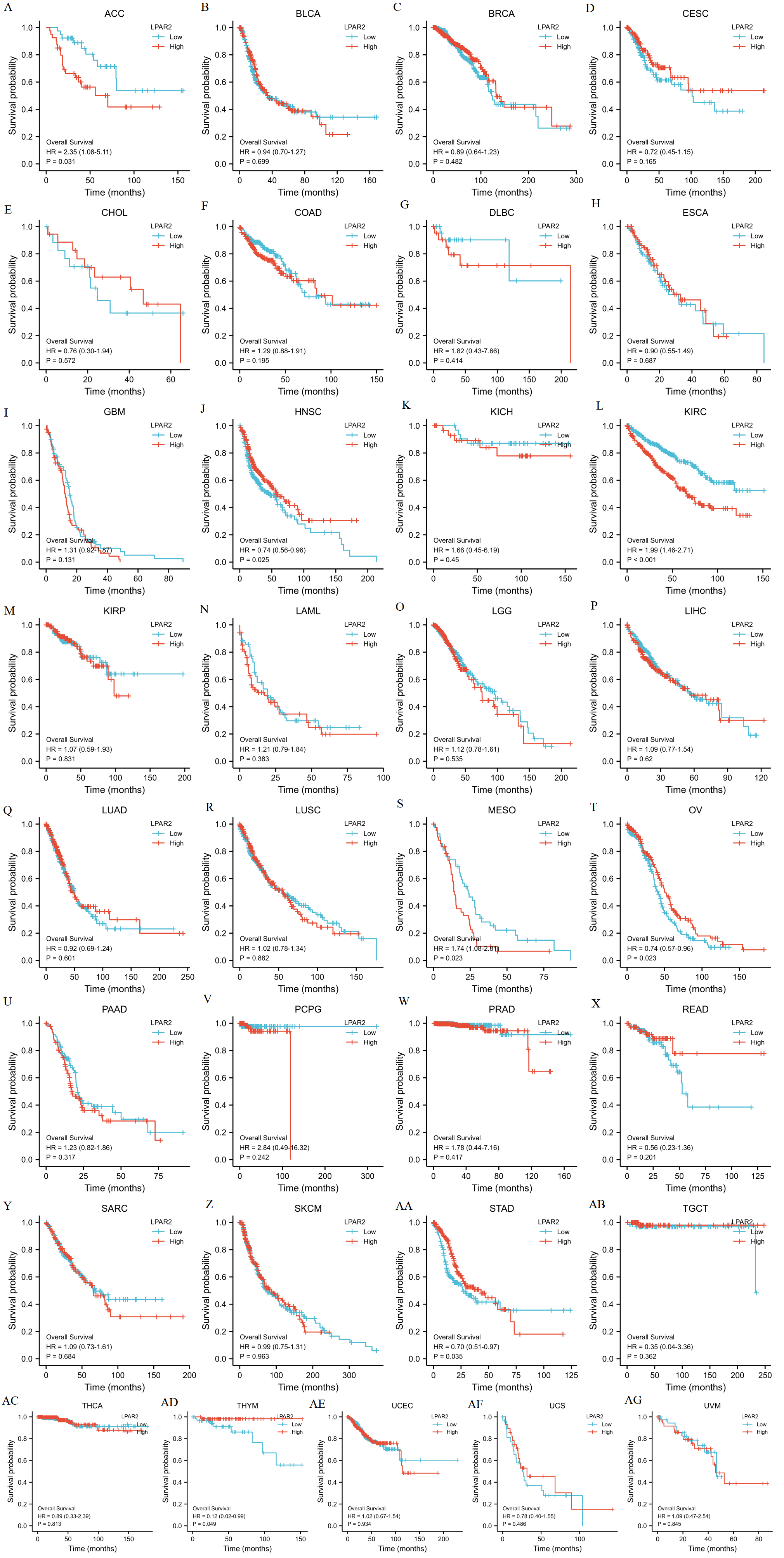

Supplement: Supplementary file 7 — Addtional file 7: Figure S4. Kaplan-Meier survival curves comparing the high and low expression of LPAR2 in different types of cancer in TCGA databases(A-AG). [file 41065_2022_229_MOESM7_ESM.tif]

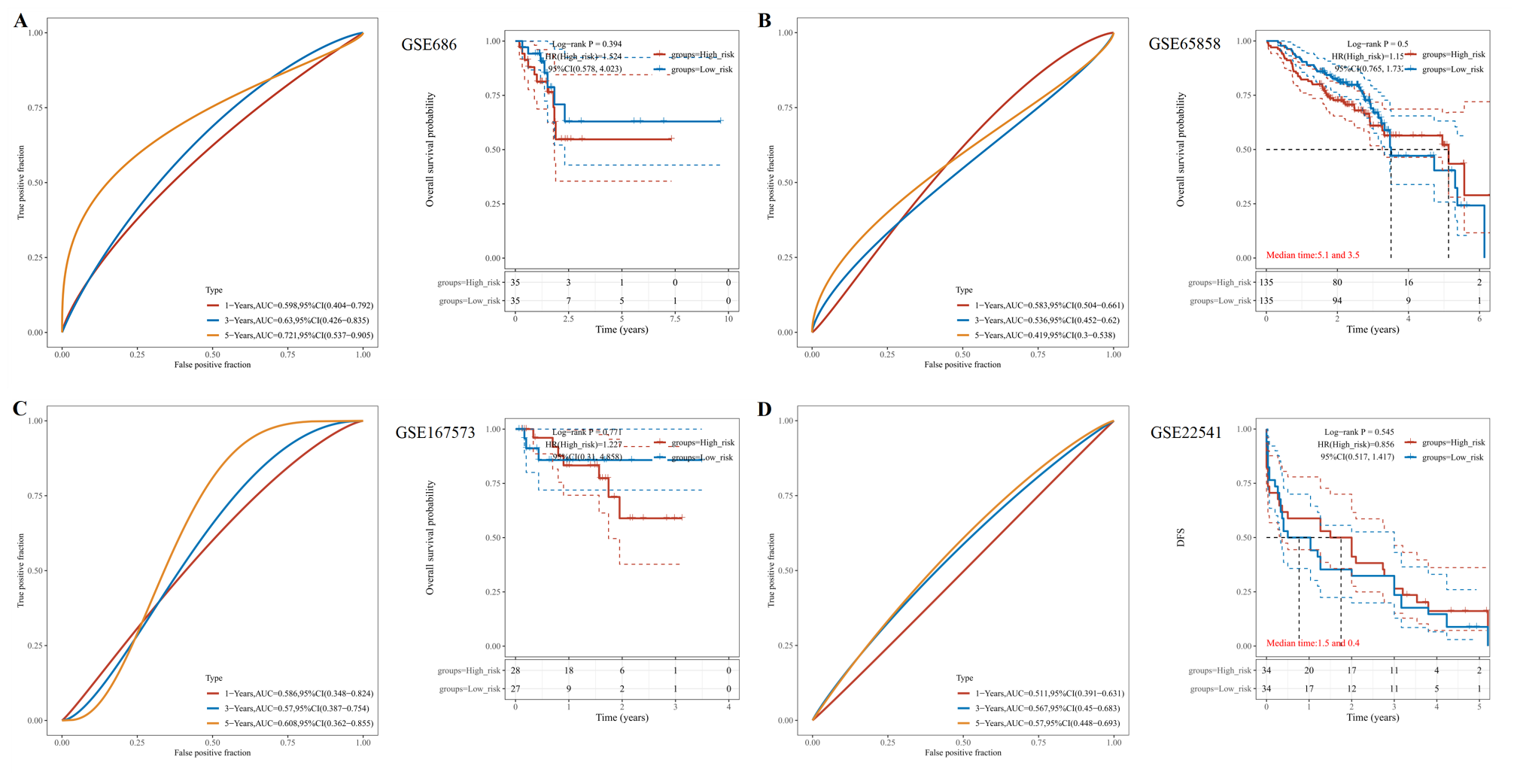

Supplement: Supplementary file 8 — Addtional file 8: Figure S5. Kaplan-Meier survival curves comparing the high and low expression of LPAR2 in HNSC and KIRC from GEO databases and the paired ROC curves of measuring the predictive value(A-D). [file 41065_2022_229_MOESM8_ESM.tif]
